# CETSA-based target engagement of taxanes as biomarkers for efficacy and resistance

**DOI:** 10.1038/s41598-019-55526-8

**Published:** 2019-12-18

**Authors:** Anette Langebäck, Smaranda Bacanu, Henriette Laursen, Lisanne Mout, Takahiro Seki, Sigrun Erkens-Schulze, Anderson Daniel Ramos, Anna Berggren, Yihai Cao, Johan Hartman, Wytske van Weerden, Jonas Bergh, Pär Nordlund, Sara Lööf

**Affiliations:** 1 Department of Oncology-Pathology, Karolinska Institutet, BioClinicum, Solna, 171 64 Sweden; 2 School of Biological Sciences, Nanyang Technological University, Singapore, 637551 Singapore; 3 Institute of Molecular and Cell Biology, A*STAR, Singapore, 138673 Singapore; 4 Department of Urology, Erasmus Medical Centre, Rotterdam, The Netherlands; 5 Department of Microbiology, Tumor and Cell Biology, Karolinska Institutet, Solna, 171 65 Sweden

**Keywords:** Breast cancer, Tumour biomarkers

## Abstract

The use of taxanes has for decades been crucial for treatment of several cancers. A major limitation of these therapies is inherent or acquired drug resistance. A key to improved outcome of taxane-based therapies is to develop tools to predict and monitor drug efficacy and resistance in the clinical setting allowing for treatment and dose stratification for individual patients. To assess treatment efficacy up to the level of drug target engagement, we have established several formats of tubulin-specific Cellular Thermal Shift Assays (CETSAs). This technique was evaluated in breast and prostate cancer models and in a cohort of breast cancer patients. Here we show that taxanes induce significant CETSA shifts in cell lines as well as in animal models including patient-derived xenograft (PDX) models. Furthermore, isothermal dose response CETSA measurements allowed for drugs to be rapidly ranked according to their reported potency. Using multidrug resistant cancer cell lines and taxane-resistant PDX models we demonstrate that CETSA can identify taxane resistance up to the level of target engagement. An imaging-based CETSA format was also established, which in principle allows for taxane target engagement to be accessed in specific cell types in complex cell mixtures. Using a highly sensitive implementation of CETSA, we measured target engagement in fine needle aspirates from breast cancer patients, revealing a range of different sensitivities. Together, our data support that CETSA is a robust tool for assessing taxane target engagement in preclinical models and clinical material and therefore should be evaluated as a prognostic tool during taxane-based therapies.

## Introduction

The use of taxanes has been a cornerstone in the treatment of cancers for several decades. Today taxanes are used in several cancer types and are a part of first line treatment in breast, non-small-cell lung cancer, castration-resistant prostate cancer, esophageal, head and neck cancers, and Kaposi sarcoma^1–3^.

Taxanes are particularly important in the treatment of both early-stage and metastatic breast cancer patients where robust improvements of overall survival have been shown^4,5^. Both paclitaxel, a substance isolated from the Pacific yew tree (*Taxus brevifolia*), and docetaxel, a more potent semisynthetic derivate of paclitaxel, derived from the European yew tree (*Taxus baccata*), are standard treatment in breast cancer. Taxanes are also used for prostate cancer patients and are currently the only type of chemotherapy used for this type of cancer. Although the mainstay therapy for metastatic prostate cancer has been hormone depletion, no effective therapy was available for patients who had progressed under androgen deprivation therapy until the introduction of docetaxel in 2004^6^. Currently the treatment landscape for patients with castrate-resistant prostate cancer (CRPC) has vastly expanded, including cabazitaxel. This novel taxane has been shown to remain effective in docetaxel-resistant CRPC patients^7^. Of note, the addition of docetaxel in parallel to androgen deprivation therapy was recently shown to increase overall survival, suggesting that an earlier switch to taxane regime might be favourable in these cases^8^.

The taxanes exert their effect by binding to β-tubulin on the luminal side of microtubules leading to the stabilization of microtubules via inhibition of depolymerization^9^. Therefore taxanes suppress microtubule dynamics, with the major mechanism for cell toxicity being the effect on the mitotic spindle, which leads to cell cycle arrest typically in G2- and M-phase, followed by cell death in rapidly dividing cells^10^. Vinca alkaloids is another family of tubulin modulators, including vinorelbine and vincristine, which act by inhibiting microtubule polymerization, also arresting cells in G2- and M-phase.

Taxanes are widely used for both early- and late-stage disease, but as for other cytotoxic cancer drugs, inherent or acquired resistance is often seen, leading to lack of efficacy or time-limited responses, and eventually progressive disease and mortality. There have been multiple mechanisms attributed to taxane resistance where a specific focus has been on the overexpression of efflux pumps, for example P-glycoprotein 1 (MDR1) or down-regulation of influx transporters^11^. The activation of efflux pumps leads to depletion of the intracellular pool of the taxane, which affects the extent of binding to β-tubulin, i.e. decrease the target engagement (TE). Attenuated TE has also been suggested to occur due to point mutations or post translational modifications of β-tubulin, modified expression levels of microtubule-associated proteins (MAPs) or altered levels of tubulin isoforms such as up-regulation of TUBB3^12–14^. However, the mechanisms for how TUBB3 overexpression overcomes cell cycle arrest remains only partially defined. Other general mechanisms for resistance to cytotoxic drugs have also been suggested to affect taxane sensitivity such as altered expression of anti-apoptotic proteins (e.g. Bcl-2 and Bcl-xL)^15,16^ and NFkB modulation^17^.

However, although several potential mechanisms and biomarkers for cellular drug efficacy and resistance have been proposed, none of these have been proven predictive for efficacy in the clinical setting. This is unfortunate since different taxanes appear to have different efficacy profiles, as well as different adverse effects, and thus biomarkers and predictive assays could potentially help in stratifying drug or drug combinations at different stages of taxane-based combination therapy.

A basic requirement for taxane efficacy is sufficient TE of β-tubulin. After the drug has reached the tumor environment, TE is mainly determined by cellular influx, efflux, and drug catabolism, yielding the effective intracellular drug concentration. During therapy the pharmacokinetic (PK) profile in the individual patient will also affect TE by determining the amount of taxanes reaching the tumour environment. To optimize PK, drug monitoring is sometimes used during taxane therapy to demonstrate appropriate serum concentrations of the drug^18^.

Considering the critical role of TE for drug efficacy, information on absolute and relative TE for different drugs in patient-derived samples could potentially help in stratification of cancer therapies with taxanes. However, direct measurements of TE in cells and tissues have previously been very challenging when no general technique for such measurements has been available. To meet this challenge, our lab has introduced the Cellular Thermal Shift Assay (CETSA), a biophysical assay which allows for TE to be measured in intact cells and tissues^19^. CETSA can directly assess drug binding at the target protein level (the protein reports) by applying the critical heating step while cells are still intact and the target protein is in its proper cellular environment. After determining overall melting behavior of a target protein, isothermal dose response (ITDR) curves can be determined with CETSA to assess relative doses needed to obtain TE. Combined with mass spectrometry (MS-CETSA) the method has allowed for comprehensive characterization of both direct drug binding, and protein interaction state changes induced by downstream and stress effects introduced by drugs.

Although CETSA is most often explored to study TE in cell cultures, single protein CETSA experiments on tissue samples have previously been done for drug treated mice, for example, in the initial CETSA study for METAP2^19^ and in several mice tissues for RIPK1^20^. Proteome wide MS-CETSA experiments have also recently been performed in multiple tissues of drug treated mice (ref. https://www.biorxiv.org/content/10.1101/500306v1).

In the present work we implemented different formats of tubulin-specific CETSA and used these to study taxane TE in cell lines of breast and prostate cancer as well as mouse xenografts. Using cognate pairs of sensitive and resistant cell lines we show that CETSA can efficiently reveal the presence of drug resistance mechanisms. Finally, using miniaturized CETSA we demonstrate that TE responses can be measured in fine needle aspirates from breast cancer patients and therefore can be used to validate tubulin CETSA as a potential prognostic biomarker for taxane sensitivity in clinical trials.

## Results

### Tubulin melting behavior and drug stabilization in western blot-based CETSA

**Figure 1:**
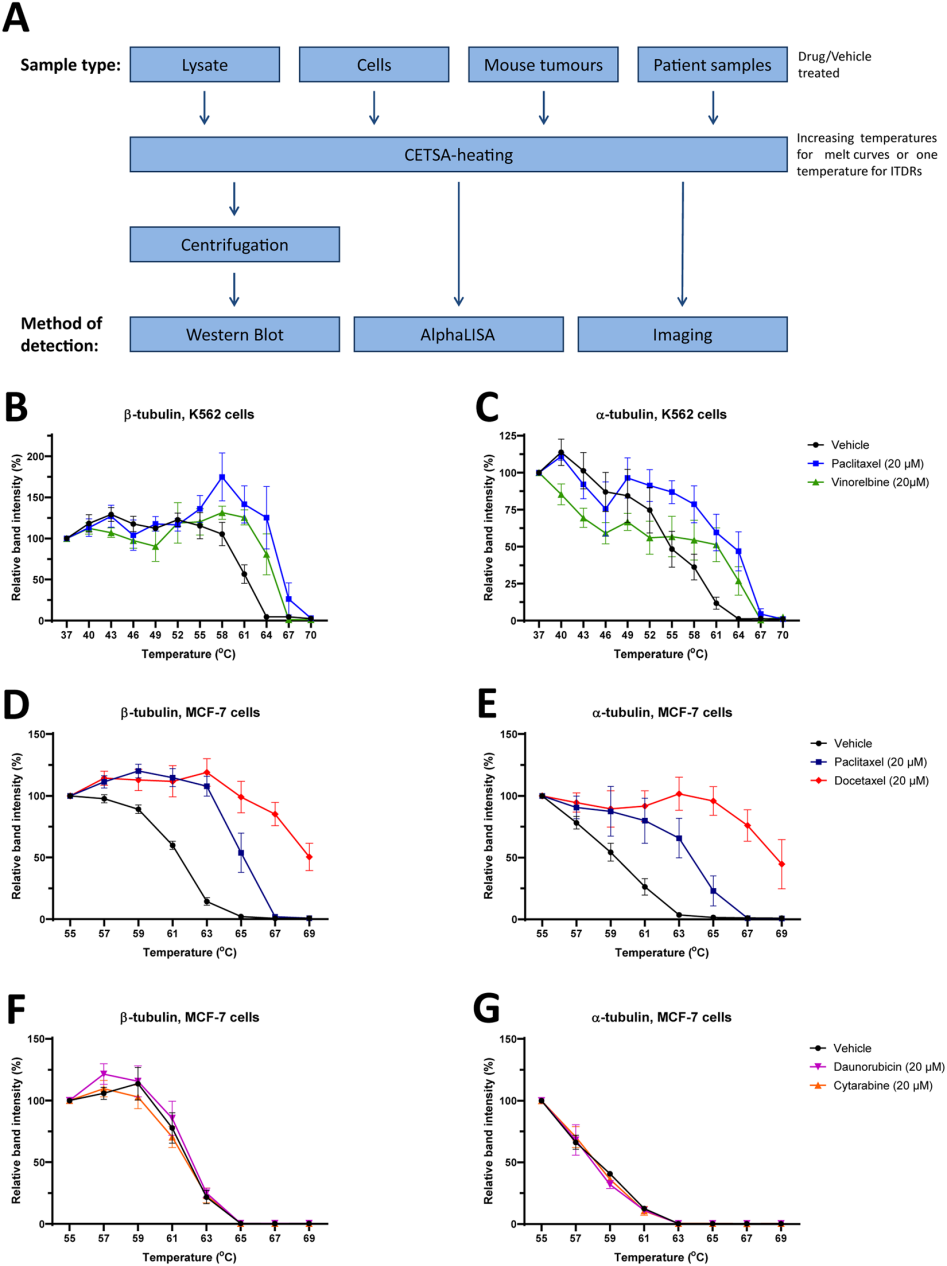
CETSA shows distinct melt curves for α- and β-tubulin that shift upon drug binding in cancer cells. Schematic overview of the CETSA method (**A**). Western blot-CETSA shows that the tubulin-binding drugs paclitaxel and vinorelbine (20 µM) produce clear shifts for β-tubulin in K562-cells (**B** and **C**). In MCF-7 cells, the two taxanes, paclitaxel and docetaxel (20 µM), produce significant CETSA shifts for both β-tubulin and α-tubulin (**D** and **E**). Daunorubicin and cytarabine (negative controls) produced no shift in both β-tubulin and α-tubulin (**F** and **G**). All data represent the mean ± S.E.M from independent experiments (n = 5–6 in **D** and **E**, and n = 3 in **B**,**C**,**F** and **G**) and are presented as a percentage of the signal detected at the lowest temperature in each melt curve.

To validate the applicability of CETSA to assess TE for tubulin-targeted drugs we implemented several CETSA formats as shown in Fig. 1A. We first developed a western blot-based assay to investigate the feasibility of tubulin-based CETSA. Initially melt curves for α- and β-tubulin were generated in K562-cells, a suspension cell line originating from myelogenous leukemia. Both α- and β-tubulins had relatively high melting temperatures, around 61–63 °C, compared to 52 °C as the average melting temperature of proteins of the human proteome^21^. The high melting temperatures of tubulins might be due to stabilizing effects of the extensive protein-protein interactions made within the microtubule polymer. This is consistent with the finding that the non-polymerized subunits of tubulin, in lysates, melt more than 10 °C earlier (Fig. S1A,B).

In addition to paclitaxel, which preferentially binds to a site on β-tubulin localized in the lumen of microtubules leading to stabilization of microtubules, we used vinorelbine, that binds to the vinca-binding domain, a site on β-tubulin distinct from the taxane-binding domain and which inhibits tubulin polymerization^22–24^. Treatment of K562-cells for 1 h with both drugs produced a significant stabilization of β-tubulin with the largest difference between vehicle and treated seen around 63 °C (Fig. 1B and Table 1). A significant shift was also observed for α-tubulin following treatment with paclitaxel (Fig. 1C and Table 1). However, no shifts were observed in cell lysates (Fig. S1A,B), consistent with the fact that drug binding is considered to occur only with intact microtubules^12^. In lysate, microtubules are depolymerized and tubulin is only present as soluble dimers. Although the drugs used bind specifically to the β-tubulin subunit, both the α- and β-subunits show shifts in CETSA melt curves (Fig. 1B–E and Table 1). We recently described the correlation of CETSA melt curves of interacting proteins and named this phenomena thermal proximity co-aggregation (TPCA)^25,26^, which is the likely explanation for the correlated shifts of the tubulin subunits. CETSA melt curves were also generated in MCF-7 cells, a commonly used model system for hormone-positive breast cancer. Paclitaxel generated clear shifts in both α- and β-tubulin in these cells, while docetaxel produced even larger shifts (Fig. 1D,E and Table 1). As negative controls we have generated melt curves for MCF-7 cells treated with the anthracycline daunorubicin, or with the pyrimidine analogue cytarabine which, as expected, produced no shifts in neither α- nor β-tubulin (Fig. 1F,G). Since the temperatures needed to generate tubulin melting are high, there might be a risk of losing cell membrane integrity during heating. We therefore tested the temperature and time dependent effects on the cell membranes in K562 and MCF-7 cells using a trypan blue assay (Fig. S1C–F). Although some cell lysis was induced above 60 °C, the majority of cells remained intact up to 63 °C. In a time dependent experiment, the cell lysis occurred mainly after 1 minute of the total 3 minutes heating time (Fig. S1E,F), which is beyond the time point when the most prominent effects on protein stability are expected (the remaining heating time is expected to mainly drive the efficient precipitation of unfolded protein).

**Table 1:** Overview of melting temperatures (T_m_), CETSA shifts (ΔT_m_),with associated standard errors and statistical significance of the measured CETSA shift for the data presented in Fig. 1B–G.

K562 cells	β-tubulin			α-tubulin		
	T_m_ (°C)	ΔT_m_ (°C)	Significance*	T_m_ (°C)	ΔT_m_ (°C)	Significance*
Vehicle	61,0 ± 0,6	—	—	54,0 ± 2,1	—	—
Paclitaxel (20 µM)	66,1 ± 0,8	5,1 ± 1,0	***	63,0 ± 1,0	9,0 ± 3,1	*
Vinorelbine (20 µM)	64,7 ± 0,8	3,7 ± 1,1	*	62,5 ± 0,5	8,5 ± 3,8	ns
**MCF-7 cells**	**T**_**m**_ **(°C)**	**ΔT**_**m**_ **(°C)**	Significance*	**T**_**m**_ **(°C)**	**ΔT**_**m**_ **(°C)**	Significance*
Vehicle	61,2 ± 0,2	—	—	58,9 ± 0,3	—	—
Paclitaxel (20 µM)	64,9 ± 0,2	3,7 ± 0,3	****	63,8 ± 0,7	4,9 ± 0,6	***
Docetaxel (20 µM)	68,9 ± 0,4	7,7 ± 0,4	****	68,5 ± 0,6	9,6 ± 0,6	****
Daunorubicin (20 µM)	61,9 ± 0,3	0,7 ± 0,4	ns	57,7 ± 0,3	−1,2 ± 0,6	ns
Cytarabine (20 µM)	61,7 ± 0,3	−0,5 ± 0,4	ns	58,1 ± 0,3	−0,7 ± 0,6	ns

*The significance of the CETSA shifts was calculated using one-way ANOVA for Tm values from independent experiments. Adjusted P-values ns P > 0.05, *P < 0.05, **P < 0.01, ***P < 0,001, ****P < 0,0001 compared to vehicle.

Importantly, when the melting temperature of tubulin in lysates is much lower than in cells, proteins from lysed cells will not be seen above 60 °C. Furthermore, no stabilizing effect by taxanes can be observed on tubulin in lysed cells when tubulin is then present only as soluble dimers, as discussed above. Therefore, the heat-induced lysis of a fraction of the cells is not likely to quantitatively affect the TE measurements with tubulin CETSA.

### Miniaturization of CETSA for β-tubulin using AlphaLISA

**Figure 2:**
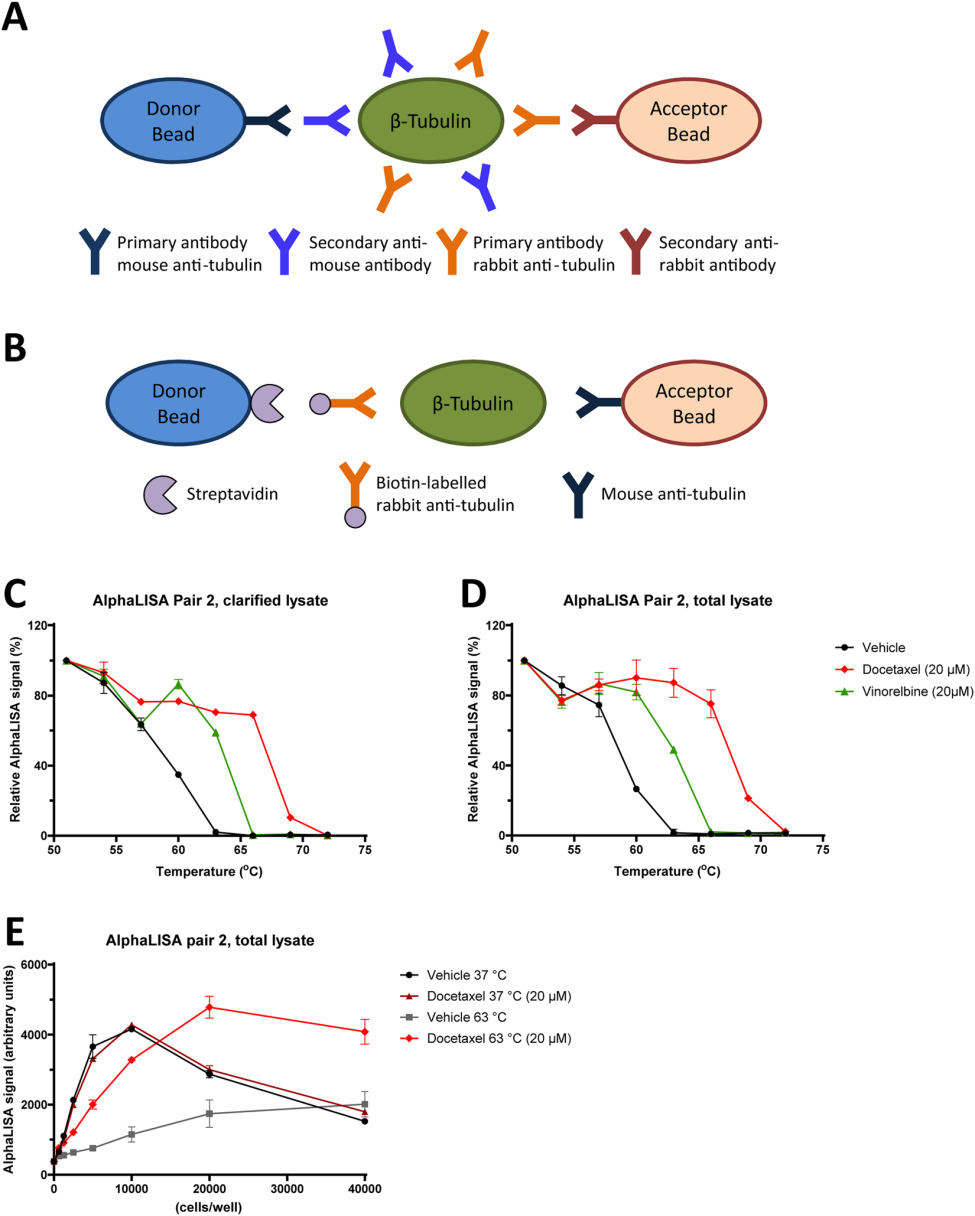
Miniaturization of the β-tubulin CETSA assay. Schematic drawing of the principle of standard and conjugated AlphaLISA (**A** and **B**). CETSA melt curves in K562 cells treated with docetaxel or vinorelbine (20 µM). β-tubulin was detected in total and clarified cell lysate with AlphaLISA pair 2 (**C** and **D**). Cell density titration for AlphaLISA pair 2 in lysate from K562 cells treated with docetaxel (20 µM) or vehicle and heated to 37 or 63 °C (**E**). The data represent the mean ± S.E.M from technical replicates and are presented as a percentage of the signal detected at the lowest temperature in each melt curve.

Since measurements on clinical samples typically are limited by the amount of available cells, and screening studies using cell lines can require high sample throughput, we established a miniaturized tubulin CETSA based on AlphaLISA (overview of method in Fig. 2A). Antibody pairs can be chosen to only detect native protein in these assays with the advantage that, after heating, centrifugation of the lysate is not needed to remove aggregated protein (Figs. 2C,D and S2B,C). Several combinations of antibodies were tested and two pairs were found to give a robust signal: pair 2 consisting of ab6046 (Abcam) in combination with sc-398937 (Santa Cruz) and pair 8 consisting of ab6046 (Abcam) and T5201 (Sigma). Both pairs could detect CETSA melt curves and target stabilization in response to docetaxel and vinorelbine with very similar shape as detected with western blot (Fig. 2C,D, Table 2, and Fig. S2B,C). Also CETSA ITDR experiments performed at 64 °C showed very similar curves with both detection methods (Fig. S2A).

**Table 2:** Overview of EC_50_ values with associated standard errors and 95% confidence intervals (CI), and statistical significance calculated for the ITDR-CETSA curves in Fig. 3A–J.

CETSA	K562-P		K562-R		
	EC50 and St. Error (µM)	95% CI	EC50 and St. Error (µM)	95% CI	Significance*
Docetaxel	0,30 ± 0,08	0,18-0,5	1,54 ± 0,25	1,10-2,20	***
Docetaxel + 0,5 µM Tariquidar	0,37 ± 0,09	0,23-0,58	0,26 ± 0,07	0,16-0,42	ns
Paclitaxel	1,55 ± 0,3	0,96-2,51	8,34 ± 1,63	5,90-11,81	***
Paclitaxel + 0,5 µM Tariquidar	1,06 ± 0,21	0,66-1,72	1,2 ± 0,27	0,80-1,78	ns
Epothilone B	0,08 ± 0,02	0,06-0,13	0,1 ± 0,01	0,08-0,14	ns
**Viab. Assay**	**(nM)**		**(nM)**		
Docetaxel	0,64 ± 0,08	0,50-0,85	150 ± 30	106-213	****
Docetaxel + 0,5 µM Tariquidar	1,64 ± 0,32	1,16-2,32	1,65 ± 0,35	1,13-2,43	ns
Paclitaxel	0,58 ± 0,14	0,37-1,02	457 ± 106	306-680	**
Paclitaxel + 0,5 µM Tariquidar	3,18 ± 0,76	2,14-4,72	3,29 ± 0,69	2,30-4,72	ns
Epothilone B	1,1 ± 0,17	0,83-1,44	1,2 ± 0,16	0,92-1,53	ns

*The significance was determined for K562-R vs. K562-P using a two-tailed T-test for EC_50_ values from independent experiments.

P-values ns P > 0.05, *P < 0.05, **P < 0.01, ***P < 0,001, ****P < 0,0001 compared to K562-P.

In order to optimize the cell numbers required for these assays different amounts of lysate were analyzed with both antibody pairs (Figs. 2E and S2D). The two pairs showed similar curves with pair 8 being somewhat more sensitive and having slightly shorter linear range. To increase the sensitivity even further, the pair 8 antibodies were directly conjugated to the AlphaLISA beads (Figs. 2B and S2E). Also a polyclonal SOD-1 antibody (R&D systems AF3418) was used in a conjugated AlphaLISA assay to function as loading control since SOD-1 is stable also at higher temperatures^27^ (Fig. S2G). The SOD1 assay had a linear range up to 500 cells (Fig. S2H). Interestingly, a time course experiment showed the stabilization of tubulin by docetaxel in K562 cells to be fully saturated already after 5 min drug treatment (Fig. S2F).

### Detection of acquired drug resistance at the TE level using CETSA

**Figure 3:**
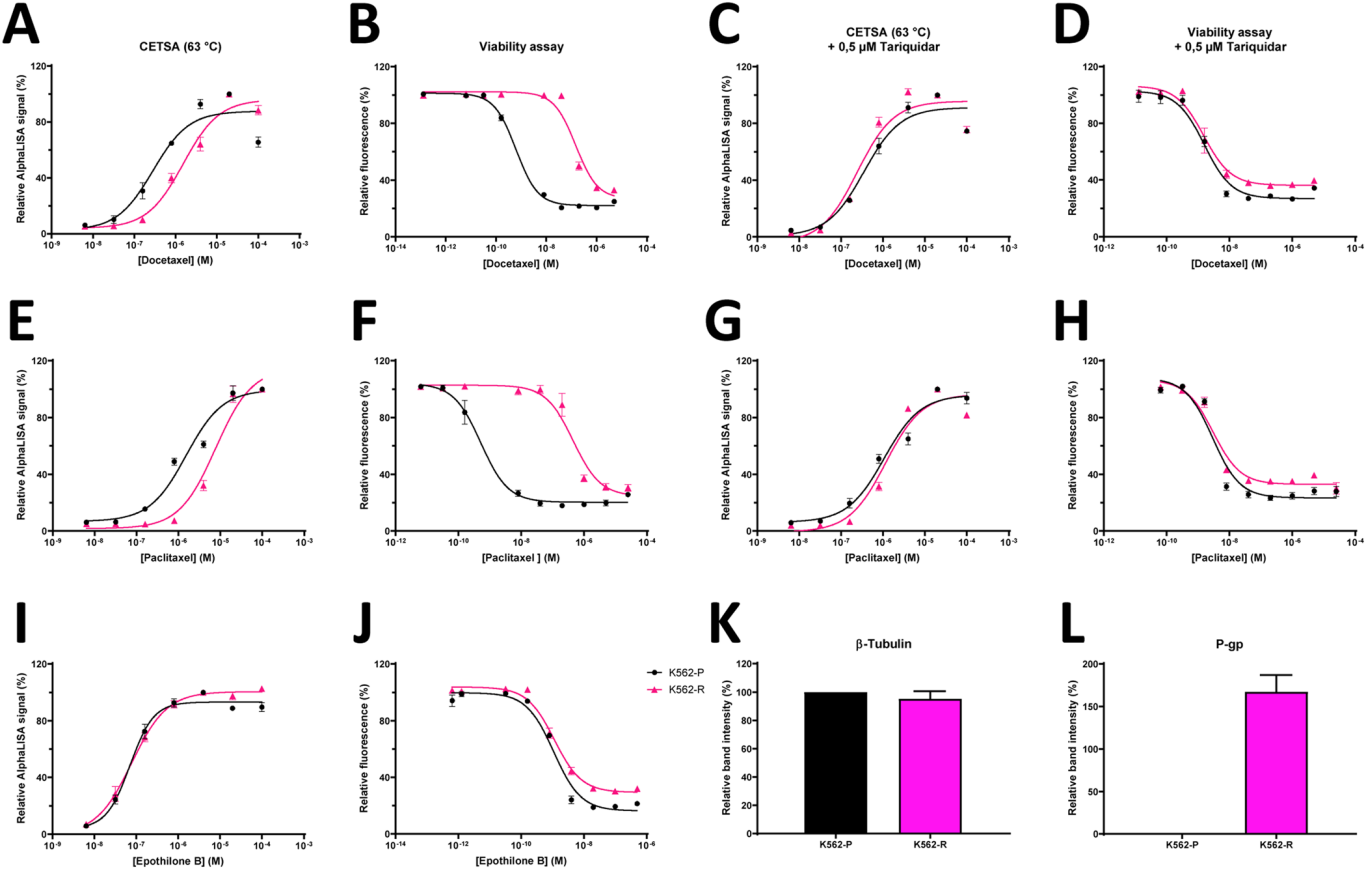
CETSA TE measurements correlate to sensitivity to taxanes and report on the mechanism of resistance. ITDR-CETSA for β-tubulin at 63 °C and viability assays in multidrug-resistant (K562-R) and the corresponding parental cells (K562-P) in response to increasing concentrations of docetaxel (**A**–**D**) or paclitaxel (**E**–**H**) and in the absence or presence of the Pgp-inhibitor tariquidar (**C,D,G,H**). ITDRs at 63 °C and viability assays performed in response to the non-Pgp-substrate epothilone B (**I** and **J**). β-tubulin was detected with AlphaLISA in the ITDR-CETSA experiments. Pgp- and β-tubulin-expression in the two cell lines was detected with western blot (**K**,**L**). ITDR-CETSA data are presented as relative to the compound concertation where maximum stabilization is achieved. Cell viability data is relative to the untreated samples. All data represent the mean ± S.E.M from independent experiments (n = 3–4).

To investigate the feasibility of using CETSA for assessing acquired drug resistance, multidrug-resistant K562-R cells were studied. The K562-R cells are previously described as vincristine-resistant due to overexpression of P-gp^28^. We used AlphaLISA as readout method for ITDR-CETSA in order to compare the dose dependence of different taxanes in K562-R and the parental counterpart K562-P after a 1 h 45 min exposure to drugs. The ITDR-CETSA data showed that K562-R cells required in the range of 5,3x higher paclitaxel or docetaxel concentrations to establish the same TE response as in the parental cells (Fig. 3A,E, and Table 2) despite no difference in the total amount of tubulin (Fig. 3K). Measurements of cell viability after 72 h using the resazurin viability assay confirmed resistance in the K562-R cells compared to K562-P (Fig. 3B,F, and Table 2). Interestingly, the differences in both cell survival and CETSA shifts were abolished by the potent P-gp-inhibitor tariquidar (Fig. 3C,D,G,H, and Table 2), directly supporting that the resistance mechanism is indeed due to up-regulation of P-glycoprotein. Furthermore, these cells were exposed to epothilone B, a tubulin targeting drug which is not a P-glycoprotein substrate. Epothilone B induced almost identical CETSA curves in K562-R as in K562-P and did not show any difference in the resazurin assay, further supporting that the observed difference between the two cells lines is mainly P-gp-dependent. Consistent with these findings, the protein levels of P-gp were found to be high in K562-R and barely detectable in K562-P as examined by western blot (Fig. 3L). Together these data illustrate that CETSA can be used to detect development of acquired drug resistance, as well as report on the mechanism of resistance in specific cases.

### Image-CETSA to monitor TE in specific cell types in heterogeneous samples

For clinical applications, the analysis of TE in individual cells is of high interest since it would facilitate assessment of TE in specific cell types within a heterogeneous cell population existing in one tumour sample. One potential way to accomplish single cell resolution in CETSA is to quantify stabilization by antibody-based cell imaging, using an antibody that specifically recognizes the folded form of the protein. We developed an imaging CETSA format where we investigated several tubulin-directed antibodies and optimized the protocols, resulting in the experimental scheme as shown in Fig. S4A. Starting from cells in suspension, after drug treatment and subsequent CETSA heating in PCR-tubes, cells were washed and collected by centrifugation before being transferred to clear bottom plates where they were fixed and stained (anti-tubulin and Hoechst). After we had developed this CETSA imaging format, two articles describing related methods were published on CETSA drug screening on adherent cells, demonstrating the feasibility of the image-based detection of target engagement^29,30^. Our method is, however, more versatile and allows for studies of clinical samples of cell suspensions such as fine needle aspirates. Moreover, our protocol allows the use of different temperatures to generate melt curves.

**Figure 4:**
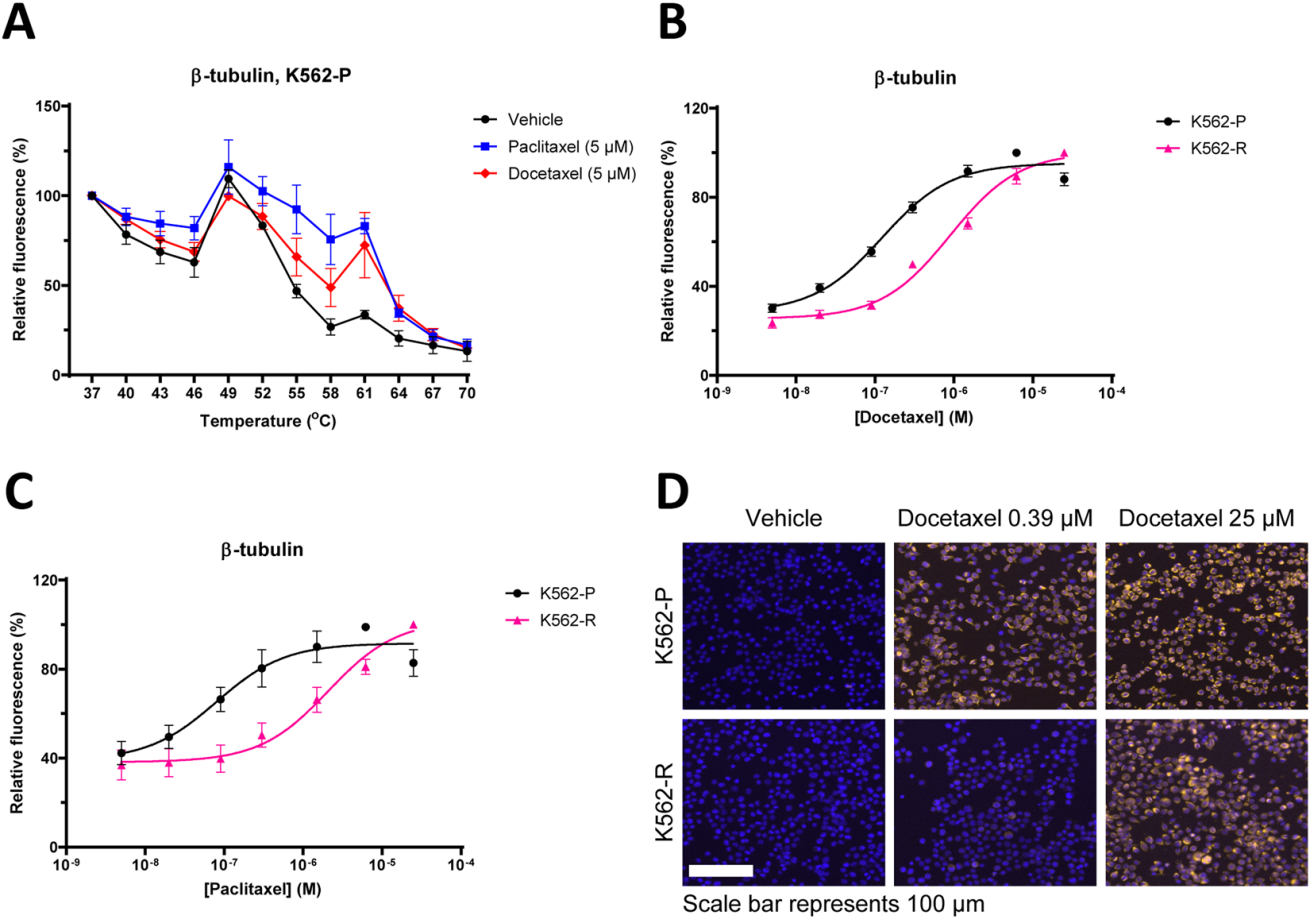
Imaging-CETSA has the potential of quantifying TE in individual cells and report on cellular resistance. Imaging-CETSA melt curves for β-tubulin in response to docetaxel and paclitaxel (5 µM) (**A**). ITDR-CETSA in multidrug-resistant (K562-R) and parental (K562-P) cells after exposure to different concentrations of docetaxel (**B**) or paclitaxel (**C**) and heating at 56 °C. Representative images of K562-P and K562-R cells treated with docetaxel or vehicle (**D**). Hoechst staining of the nuclei is shown in blue while β-tubulin staining is shown in yellow. All data represent the mean  ±S.E.M from independent experiments (n = 3–4) and are presented as a percentage of the signal detected at the lowest temperature in each melt curve (**A**) or maximum stabilization detected in each series (**B** and **C**).

To test our tubulin Image-CETSA implementation for assessing drug efficacy, we first generated melt curves by exposing K562 cells to different temperatures after incubation with and without docetaxel or paclitaxel (Fig. 4A and Table 3). The Image-CETSA melt curves were similar to melt curves produced by western blot CETSA from the same samples and both drugs have apparent stabilization (Figs. S4B and 4A). We then performed ITDR-CETSA experiments, showing that the imaging protocol was able to generate dose response curves (Figs. S4C and 4B). ITDRs with docetaxel were also performed in the resistant K562-R cells comparing them with their parental counterparts K562-P. A shift towards a higher EC_50_ value was observed in the ITDR-CETSA curve corresponding to K562-R cells for both paclitaxel and docetaxel, supporting that Image-CETSA can indeed be used to monitor drug sensitivity (Fig. 4B,C and Table 3). In Fig. 4D, representative images show that Image-CETSA in principle can reveal TE information in individual cells and should be applicable to monitor TE in individual cell types in heterogeneous samples.

**Table 3:** Overview of EC_50_ values with associated standard errors and 95% confidence intervals (CI), and statistical significance calculated for the ITDR-CETSA curves in Fig. 4B,C.

Docetaxel	Imaging-CETSA		
	EC50 and St. Error (µM)	95% CI	Significance*
K562-P	0,12 ± 0,02	0,09-0,16	—
K562-R	0,83 ± 0,12	0,60-1,17	**
**Paclitaxel**	**EC50 and St. Error (µM)**	**95% CI**	Significance*
K562-P	0,08 ± 0,04	0,03-0,20	—
K562-R	2,00 ± 0,83	0,73-5,41	**

*The significance was determined for K562-R vs. K562-P using a two-tailed T-test for EC_50_ values from independent experiments.

P-values ns P > 0.05, *P < 0.05, **P < 0.01, ***P < 0,001, ****P < 0,0001 compared to K562-P.

### Taxane TE in mouse xenografts in *ex vivo* and *in vivo* settings

**Figure 5:**
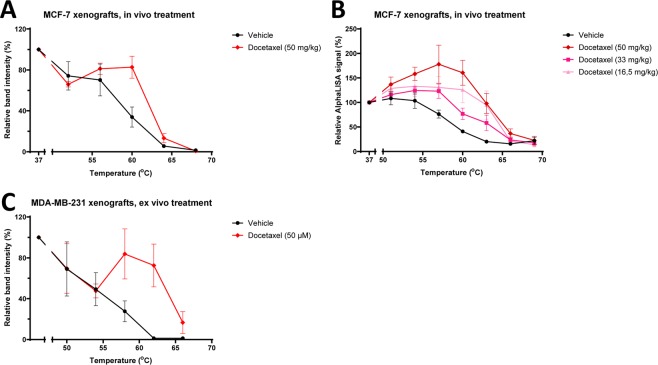
Docetaxel produces CETSA shifts for β-tubulin in both *in vivo* and *ex vivo* mice models. SCID-mice bearing MCF-7 xenograft tumours were treated *in vivo* for 30 min with docetaxel at a dose of 50 mg/kg before being sacrificed and the tumours taken for β-tubulin analysis with western blot-CETSA (**A**). SCID-mice bearing MCF-7 xenograft tumours were treated *in vivo* with different doses of docetaxel and samples were analysed with AlphaLISA (**B**). Pieces of MDA-MB-231 xenografts were treated *ex vivo* with Docetaxel (50 µM) before β-tubulin analysis with western blot-CETSA (**C**). All data represent the mean  ±S.E.M from different tumours in each condition (n = 2–3 in A, and n = 4 in **B** and **C**) and are presented as a percentage of the signal detected at the lowest temperature in each melt curve.

An advantage of CETSA is that the same measurement principle for target engagement can be used in both cell lines and tissues samples. To explore the use of tubulin CETSA in animal models, we used xenografts in mice for *ex vivo* and *in vivo* treatment. First, mice with MCF-7 derived xenografts were injected *i.v*. with drug or vehicle 30 min before euthanasia, directly followed by tumor sampling and preparation for CETSA analysis. In this first experiment a dose of 50 mg/kg was used, which is equivalent to 150 mg/m^2^ for humans^31^, a dose that is somewhat higher than routinely used in the clinic. A significant stabilization of β-tubulin was observed for β-tubulin at 60 °C (Fig. 5A and Table 4).

**Table 4:** Overview of the statistical significance of the measured CETSA shift for the data presented in Fig. 5A–C.

MCF-7 Xenografts	β-tubulin
*in vivo* (60 °C)	Significance*
Vehicle	—
Docetaxel (50 mg/kg)	*
*in vivo* (60 °C)	Significance*
Vehicle	
Docetaxel (16,5 mg/kg)	*
Docetaxel (33 mg/kg)	ns
Docetaxel (50 mg/kg)	**
*ex vivo* (62 °C)	Significance*
Vehicle	—
Docetaxel (50 µM)	*

*The significance of the CETSA shifts was calculated using one-way ANOVA.

Adjusted P-values ns P > 0.05, *P < 0.05, **P < 0.01, ***P < 0,001, ****P < 0,0001 compared to vehicle.

In a second experiment we therefore repeated the treatment with 50 mg/kg docetaxel in mice bearing MCF-7 xenografts and included two additional docetaxel doses (33 mg/kg and 16,5 mg/kg) and two additional temperatures for the melt curves. This is equivalent to human doses of 100 mg/m^2^ and 50 mg/m^2^, which are commonly used in monotherapy and combination treatment respectively. A significant stabilization of tubulin was observed also with lower doses of docetaxel, demonstrating that CETSA-based TE can potentially be detected at clinically relevant doses (Fig. 5B and Table 4).

For comparing local tumor cell effects of different drugs, an *ex vivo* setting experiment is preferred, since this format would allow to evaluate target binding of multiple drugs in e.g. the same patient biopsy. To test this setting and an additional xenograft model, *ex vivo* experiments were performed in MDA-MB-231 (triple negative breast cancer cell line) derived xenograft tumors. *Ex vivo* exposure to 50 µM docetaxel also showed a very prominent shift, albeit with larger standard deviations than in the *in vivo* treated MCF-7 samples (Fig. 5C and Table 4). Possibly, the larger standard deviations could be due to poorer drug penetration in the solid tumor MDA-MB-231 pieces in the *ex vivo* setting and indicate that analysis of tissue samples with *ex vivo* drug treatment should be done using cell suspensions obtained by digesting solid biopsies, rather than by treating pieces. Alternatively, to avoid digestion of tumor tissue, a process that could potentially affect cell characteristics, the tissue could be freshly sliced using a vibratome before incubating with drug.

### Characterization of resistance in prostate cancer PDX models

**Figure 6:**
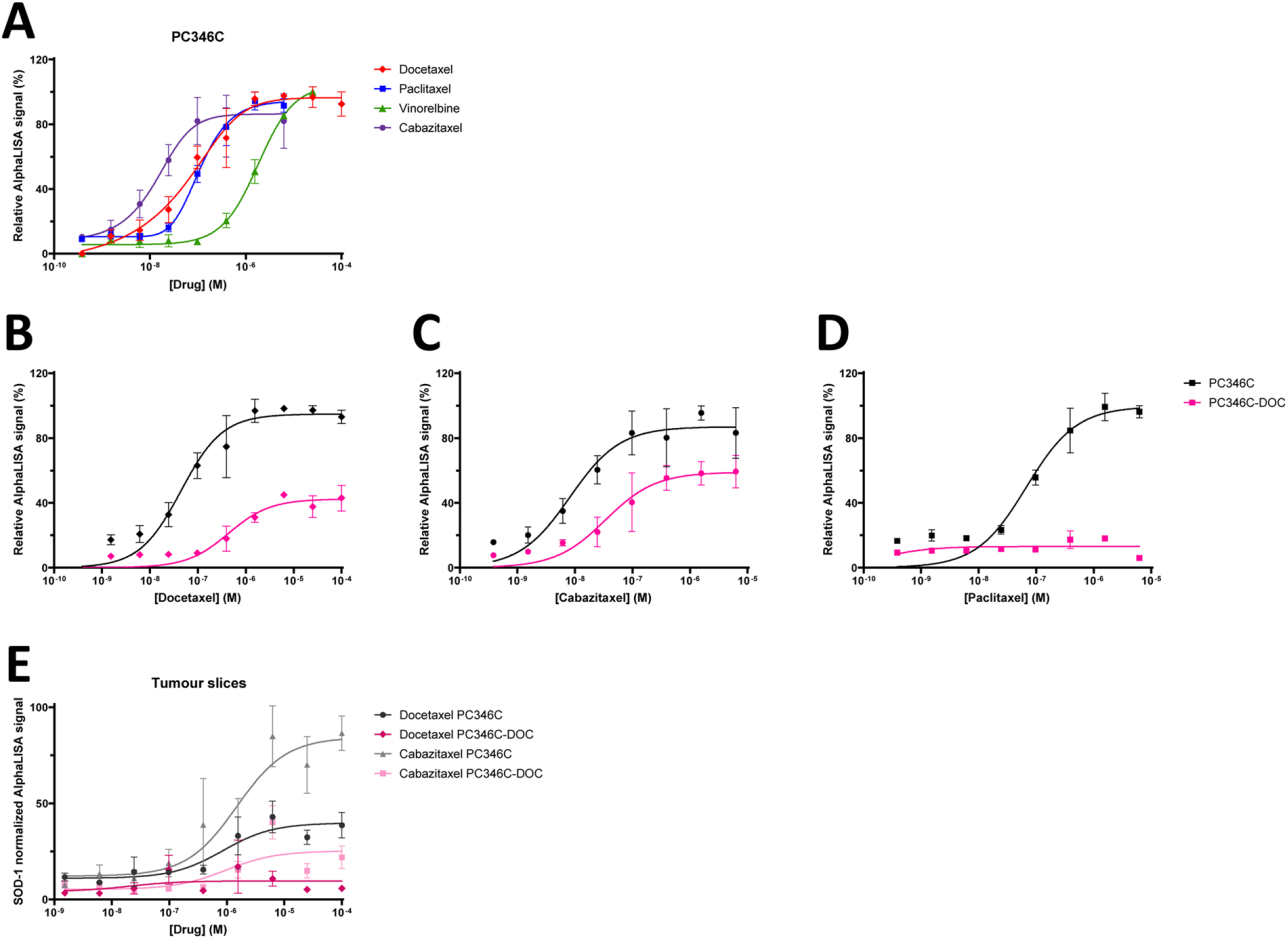
CETSA TE levels correlate with sensitivity to taxanes and reported resistance mechanisms in mouse PDX models of prostate cancer. PDX-derived cell line PC346C was treated with different concentrations of tubulin-binding drugs and β-tubulin TE was analysed with AlphaLISA (**A**) PC346C cells and the corresponding resistant cell line PC346C-DOC were treated with different concentrations of taxanes (**B**–**D**). Tumours from the PDX model PC346C and the resistant counterpart PC346C-DOC were treated *ex vivo* with increasing doses of docetaxel and cabazitaxel (**E**). Data from tumours slices were normalized to SOD-1 levels. All data represent the mean  ±S.E.M from either different tumors in each condition (**E**) or from independent experiments (n = 3 in **A–D**).

As previously mentioned, an attractive setting to predict clinical outcome of drug treatment is to examine drug response *ex vivo* in biopsy samples. To investigate if drug TE measured with CETSA in *ex vivo* experiments could predict *in vivo* biology of the same drug, we performed experiments using patient-derived xenograft (PDX) based models of tumour drug resistance in castration-resistant prostate cancer. To study taxane sensitivity in resistant models we used two PDX models, PC346C and PC339, and their docetaxel-resistant counterparts PC346C-DOC and PC339-DOC, previously described by de Morrée *et al*.^32^. Relevant details regarding the abovementioned PDX models are summarized in Table S1A. We first compared the effect of docetaxel, cabazitaxel, paclitaxel, and vinorelbine in cell lines corresponding to the PDX models PC346C and PC339. We found a pronounced difference in potency between the drugs in both cell lines with cabazitaxel as the most potent drug, followed by docetaxel, paclitaxel, and vinorelbine as the least potent drug (Figs. 6A, S5A and Table S1B). The observed ranking correlates with previous studies showing cabazitaxel is more efficient than docetaxel in inhibiting cell proliferation and suppression of microtubule dynamics^33^.

Studies of the PC346C and PC346C-DOC cell lines support that the taxane-resistant cells have attenuated TE in response to docetaxel, cabazitaxel, and paclitaxel (Fig. 6B-D and Table S1D) when both EC50s and maximum response levels are affected, reiterating the published *in vivo* results showing that taxane uptake is strongly impaired in the PC346C-DOC model.

PC346C-DOC express less of the influx transporter SLCO1B3, as compared to the parental counterpart PC346C, which is shown to result in almost fully depleted intratumoural taxane levels^34^. The effect of cabazitaxel on TE was more pronounced than that of docetaxel in both PC364C and PC346C-DOC tumor slices, which is in line with its reported higher efficacy^35^.

Similar studies of PC339C and PC339C-DOC do not show any significant difference between the cell lines, consistent with that processes effecting residual target engagement are not altered in this model.

Subsequently, *ex vivo* treatments of PC346C and PC339 PDX tumors (freshly sliced using a vibratome) with increasing concentrations of docetaxel and cabazitaxel were performed. Standard deviations were high for these experiments for technical reasons, as discussed below, but the results support that maximum β-tubulin TE in PC346C-DOC compared to the parental counterpart PC346C after both docetaxel and cabazitaxel treatment (Fig. 6E) is changed in a similar manner as in the cell lines. In contrast, PC339 and the docetaxel resistant variant PC339-DOC did not show effects on TE (Fig. S5E). Previous data show PC339-DOC to remain sensitive to cabazitaxel and that the docetaxel resistance in this model is possible to overcome by increased dosing of docetaxel^32^. These results support that the mechanism of resistance in PC339-DOC is to be downstream of, or alternatively bypassing, target engagement. Taken together, our data support that CETSA TE studies can differentiate between sensitive and resistant tumors in the cases where taxane resistance occurs up to the level of TE, for example in the case of altered expression of membrane transporters.

### Drug TE measurements in fine needle aspirates from breast cancer patients

**Figure 7:**
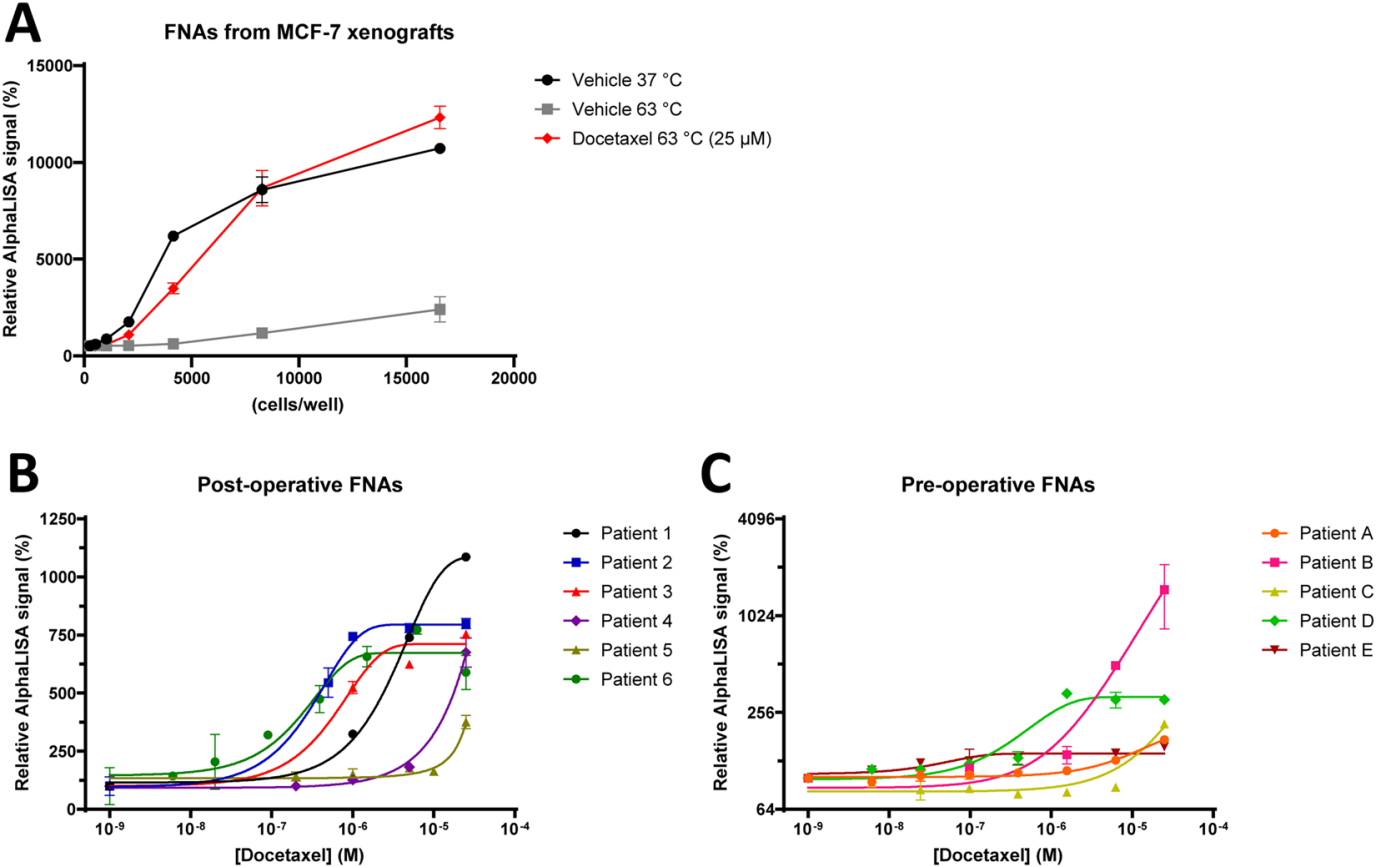
CETSA for assessing TE for taxanes in *ex vivo*-treated breast cancer patient FNAs. Fine needle aspirates (FNAs) were taken from freshly removed MCF-7 xenografts and treated with docetaxel (25 µM) for 15 min before CETSA-heating at 37 or 63 °C. β-tubulin for different lysate concentrations was analysed with AlphaLISA (**A**). Fine needle biopsies from surgically removed tumours (**B**) or directly from patients (**C**) were treated with different concentrations of docetaxel for 15 min before CETSA-heating at 63 °C and β-tubulin analysis with AlphaLISA. The data represent the mean ± S.E.M from technical replicates and are presented as a percentage of the signal detected in the vehicle-treated samples.

As shown above in several model systems, tubulin CETSA for taxanes generally correlates with drug sensitivity. To establish and evaluate the method for clinical samples we used fine needle aspirates (FNAs), since previous data indicates that measurements in cell suspensions tend to have lower variation than tissue pieces and that tumor cells are highly enriched in FNA samples^36^. We first tested the amenability of using FNAs by taking such samples from mouse xenografts. CETSA stabilization upon *ex vivo* treatment with 25 µM docetaxel of FNAs from MCF-7 xenografts was detected by AlphaLISA and shown to be linear for several cell concentrations (Fig. 7A).

Next, the applicability of CETSA for patient material was assessed by performing experiments on FNAs collected from surgically resected primary tumors from breast cancer patients that have not received any previous treatment. All FNAs were exposed to docetaxel for 15 min, an incubation time that was shown to be sufficient to give full stabilization (Fig. S2F) and yet short enough to have minimal impact on cell viability as well as minimizing potential effect of transcription or other alterations that might occur in cells after being removed from their original environment. A major challenge with this approach was however the low cell number and viability typically obtained from the FNAs, although this varied very significantly between samples (Fig. S6). FNAs containing enough material (at least 10 000 live cells per experiment for detection with conjugated beads, and 100 000 per experiment for detection with standard AlphaLISA) were subjected to docetaxel treatment and subsequent CETSA analysis. Clear stabilization of β-tubulin could be detected in most samples analyzed and variability was acceptable between technical replicates. In cases where a sufficient number of viable cells were obtained, FNA samples were incubated with several doses of docetaxel. Surprisingly, these experiments revealed large differences at TE level between patients, up to 100 times (Fig. 7B and Table S2).

FNAs were also collected by cytological aspiration directly from the untreated primary tumours of breast cancer patients. In this case, the viability of the cells was somewhat higher with a mean of 43% cell viability compared to 16% in the FNAs from surgically removed tumors (Fig. S6). This improvement in cell viability may have been the result of the shorter time between FNA collection and performing the CETSA analysis. When FNAs were collected from surgically removed tumours this was typically done at least 1 hour after time of ischemia, which could have contributed to the differences in viability. The assay quality was, however, similar when analyzing samples taken directly from patients and from surgically removed tumours. Again, samples showed a dose dependent response to docetaxel but the efficacy varied between patients (Fig. 7C). K562 cells were treated and analyzed in parallel in each experiment as a technical control of the experiment and for confirming the reproducibility of the AlphaLISA assay. The K562 control experiments resulted in ITDR-CETSA curves for docetaxel with very similar EC50 values for all tested samples (data not shown).

## Discussion

In the present work we establish different formats of CETSA to assess target engagement of taxanes, and other tubulin directed drugs, to microtubules in cells and tissues, including breast cancer patient FNAs. The different CETSA formats give consistent measurements of melting behavior and ITDRs, although some quantitative differences are seen in melting curve shapes. We show that melt curves are stabilized by several tubulin modulators binding at both the vinca and the taxane site on β-tubulin. The very stabilization by vinorelbine is surprising and indicates that this family of drugs, in addition to inhibiting tubulin polymerization, also have a dramatic effect in intrinsic microtubule stability, which might add significantly to their cytotoxic mechanism. The optimized AlphaLISA assays provide a miniaturized and sensitive format allowing for measurements using relatively small number of cells (1000 cells per measurement point). Miniaturized and sensitive CETSA measurements are particularly valuable when many measurements points (replicates, doses, time points, etc.) are assessed in model studies, and sensitivity is essential for measurements on biopsy material where low cell number is often the limiting factor. The imaging CETSA implementation provides an interesting alternative, when different cell types can be selected from images, and TE can in principle be assessed in each cell type (although this was not directly tested in this study).

A specific challenge of the tubulin specific assay is the unusually high melting temperature of tubulin in intact cells, being in the range where some cells are prone to heat induced lysis. Fortunately, tubulins are depolymerized when cells lyse and do not bind taxanes. Furthermore, tubulin in lysed cells melt some 10 °C earlier than in intact cells, so no protein from lysed cells is detected at the temperature used for most of the ITDRs (63 °C).

Most studies of cancer drug sensitivity is done using cell viability assays with durations of 48–72 h, but such assays are typically hard to establish in reproducible formats for material from solid tumors. Also, different cell programs and distribution of different cancer cell clones in the sample can change very significantly during extended culturing, as compared to the situation in the tumor. An advantage with the *ex vivo* CETSA assay is that it allows for rapid measurement of TE, where fresh tumor samples are incubated with different drugs for short time periods (15–120 minutes), during which cell programs and cell clone distributions are less affected. On the other hand, CETSA only assesses cellular responses up to the target engagement level. For tubulin-directed drugs, however, several mechanisms proposed so far for drug resistance include the level of effective intracellular drug concentrations (through changes in influx, efflux, or catabolism) as well as modulation of tubulin structure (e.g. tubulin mutations or overexpression of beta-III-tubulin)^12–14^, which should be directly accessible with CETSA measurements.

In support that CETSA-derived measurement of tubulin binding correlates well with cell toxicity we show that CETSA ITDRs reflect known ranking of toxicities of different tubulin binding drugs in cell lines. When toxicity of tubulin inhibition is primarily expected in M-phase, this suggests that drug binding and buildup of effective drug concentration in other cell cycle phases, that dominate our samples, correlates well with the situation in M-phase. Importantly, CETSA directly reflects the difference between parental and resistant cells as shown for the multidrug resistant K562 cells as well as for PC346C-DOC taxane-resistant prostate cancer.

In the PC339-DOC PDX model TE was retained, consistent with the known increased drug accumulation of cabazitaxel in the resistant cells. This supports a bypass, or downstream mechanism of acquired resistance for this PDX model which remains to be characterized. This illustrates that even in cases were CETSA reveals TE not to be affected, such information will be valuable for dissecting resistance mechanisms for cancer drugs, since CETSA captures both effective intracellular drug concentrations and target modifications. In cases where TE is not changed in resistant cells, focus can instead be put on discovering downstream mechanisms for resistance. The mass-spectrometry implementation of CETSA (MS-CETSA) now provides a novel mean to dissect downstream/bypass mechanisms for drug resistance involving modulations of protein interaction states^26^.

Clinical evaluation of tubulin CETSA is outside the scope of this work and remains to be done for the validation of the usefulness of the assay to predict clinical outcome and to stratify drug selection. The present work does, to the best of our knowledge, for the first time present CETSA data for patient samples revealing variations in dose response. When tubulin CETSA data show correlation with drug toxicity in models it has the potential to be useful as an early prognostic biomarker to detect the emergence of resistance for different drugs up to the TE level in patient biopsies, where the cross resistance to alternative tubulin directed therapies can also be accessed. It is likely that drug targets for many other clinical drugs are similarly applicable for CETSA and that the resistance of other classes of drugs in combination therapies can be accessed in parallel with tubulin-specific acquired resistance. In the clinical situation, it has previously been difficult to pin down acquired resistance for individual drugs in combination regimes, except in cases where information on specific mutational patterns leading to target protein modifications has been available.

In conclusion, we have established and validated several protocols for measuring tubulin-specific CETSA of taxanes in cell lines and mouse models, demonstrating that drug effects up to the level of TE can be rapidly assessed in cells and tissues. Correlation of CETSA-based detection of TE with cell toxicity in several of the tested models support that CETSA measurements can provide a novel and valuable alternative in the clinical setting to rapidly generate actionable information for therapeutic decisions. Experiments on FNAs from breast cancer patients demonstrate that CETSA TE can indeed be measured in some patient samples with sufficient accuracy in the *ex vivo* setting. The relatively broad range of responses on the TE level seen in these non-treated patients, suggest varying drug sensitivity and potentially that initial resistance can be detected in patients. To rapidly assess the relative efficacy of several tubulin directed drugs could therefore help in stratification of next line therapies. However, in spite of the high sensitivity of the miniaturized assays, for many patients the number of viable cells were not sufficient from a single FNA (Fig. S6) suggesting that e.g. core biopsies, and more sensitive detection methods are needed for CETSA measurements in a majority of patients.

## Materials and Methods

### Drugs

Docetaxel (S1148), paclitaxel (S1150), vinorelbine (S4269), epothilone B (S1364), daunorubicin (S3035), and cytarabine (S1648) were obtained from Selleckchem. All drugs were solubilized in DMSO at a concentration of 50 mM and stored frozen until use. Drugs were generally diluted 4 times in DMSO before further dilution in HBSS or media.

### Cell culture

All cell lines were cultured in 5% CO_2_ and 37 °C. K562 (ATCC-CCL-243) was grown in RPMI (Sigma R8768), SK-BR-3 (ATTCC-HTB-30) in McCoy’s 5a media (Sigma), and MCF-7 (ATCC-HTB-22) and MDA-MB-231 (ATCC-HTB-26) were grown in DMEM high glucose (Sigma D6429). All media were supplemented with 10% heat inactivated fetal bovine serum (FBS) (Gibco 10500-064) and antibiotic-antimycotic (Life technologies 15240-062). Multidrug-resistant and parental K562 cells were kindly provided by Sören Lehmann and Christer Paul at the Karolinska Institute. Cells were passaged every second to third day. For adherent cells passaging included washing the cells in HBSS (Life technologies 14175-053 -CaCl_2_, -MgCl_2_) and detaching in TrypLE (Life Technologies 12563-029). The PC346C and PC339C cell lines were cultured in prostate growth media (PGM) as described in^37^ and the docetaxel-resistant counterparts were cultured in the presence of 0.1 nM docetaxel. The multidrug-resistant K562-cells were incubated for 72–96 hours with 150 nM vincristine (or DMSO for the parental cells) once a week.

### CETSA melt curves and ITDR-CETSA with intact cells

Cells were harvested, washed, and diluted in HBSS (Life technologies 14025100 + CaCl_2_, + MgCl_2_) to 4 million cells/ml for western blot and 3,33 million cells/ml for AlphaLISA. The cell suspension was mixed with drug or vehicle before aliquoting into PCR-tubes and kept at 37 °C for 1 h unless otherwise indicated. During longer incubations, tubes were rotated every 20 min. CETSA heating for 3 min, unless otherwise stated, was performed in a Veriti Thermal cycler (Applied Biosystems), either at a range of temperatures for melt curves or at 63 °C for ITDRs unless otherwise specified. Cells were subsequently lysed by three rounds of freeze-thawing by alternating exposure of the samples to liquid nitrogen and 20 °C in a PCR-machine. For western blot experiments the aggregated proteins were removed by centrifugation at 20 000 g for 20 min at 4 °C and the supernatant was collected. Samples were either analyzed directly or stored at −80°.

### CETSA melt curves with cell lysate

Cells were harvested, washed, and diluted in HBSS (Life technologies 14025100 + CaCl_2_, + MgCl_2_) to 40 million cells/ml and freeze-thawed three times, as described above, with vortexing in between for proper cell lysis. The high cell density was used in order to obtain a protein concentration more similar to that inside intact cells. The lysates were centrifuged at 20 000 g for 20 min at 4 °C, the supernatant collected, and the clarified lysate was stored at −80° until use. For the experiments, the lysate was mixed with drug or vehicle and incubated in PCR-tubes for 10 min at RT. CETSA heating and centrifugation were performed as described above. Before western blot analysis the samples were diluted to 5x in HBSS before mixing with the western blot loading buffer in order to get the same protein concentration in the samples as used for the intact-cell experiments.

### Western blot

Samples were mixed with NuPage loading buffer consisting of NuPage LDS sample buffer (Life technologies NP008) and reducing agent (Life technologies NP009) and vortexed. Proteins were separated on a Bis-Tris 4–12% polyacrylamide midi gel (Invitrogen WG1403BX10) for 45–50 min at 200 mV. Directly after the run the gels were washed in deionized water and transferred to nitrocellulose membranes using the iBlot 2 system (Invitrogen) and iBlot 2 NC Regular stacks (Invitrogen IB23001). Membranes were washed for 10 min in TBS with 0,05% Tween 20 (Medicago 09-7510-100) (TBS-T) and blocked in 5% (w/v) non-fat milk (Semper AB) in TBS-T for 1 h before incubation with primary antibody over night at 4 °C with gentle shaking. After washing in TBS-T for 5 × 5 min, membranes were exposed to secondary antibodies for 1 h, washed again 5 × 5 min in TBS-T and developed using Clarity Western ECL Substrate (BioRad #170-5061). The chemiluminescent signal was detected using the ChemiDoc^TM^ XRS + imaging system from BioRad and band intensities were quantified using ImageLab^TM^ software (BioRad).

Primary antibodies used were T5201 from Sigma for β-tubulin, sc-32293 from Santa Cruz for α-tubulin, and HPA001401 from Sigma or sc-11407 from Santa Cruz for SOD-1. When comparing western blot with Image-CETSA the β-tubulin antibody #2128 from Cell Signalling was used since that is the same clone (9F3) as the Alexa Fluor 488 conjugated antibody #3623 that was used for Image-CETSA.

Secondary antibodies used were either sc-2055 from Santa Cruz or W402B from Promega for mouse and sc-2374 from Santa Cruz or W401B from Promega for rabbit. All antibodies were diluted in 5% (w/v) non-fat milk in TBS-T.

### Trypan blue exclusion

In order to monitor membrane integrity after heating, cells were harvested according to the same protocol as for the CETSA experiments (see above) and heated in the Veriti Thermal cycler according to the indicated temperatures and time points. After heating, a 10 µl aliquot was immediately taken and mixed with 10 µl 0.4% Trypan Blue stain (Gibco/Life Technologies 15250-061). 10 µl of this mix was directly loaded on a counting slide (BioRad #145-011) and read in an automated cell counter (BioRad TC20).

### Standard AlphaLISA assay

For detection with AlphaLISA, 3 µl of each sample was loaded in duplicates in an AlphaPlate (Perkin Elmer 6008350) followed by addition of 2 µl antibody mix. Two different combinations of antibodies were used: either the mouse-anti-tubulin antibody sc-398937 (raised against amino-acids 209–305 within an internal region of human beta4-tubulin, Santa Cruz) in combination with the rabbit-anti-tubulin antibody ab6046 (raised against a synthetic peptice corresponding to human beta tubulin amino-acids 1–100, Abcam) referred to pair 2, or pair 8 where the mouse antibody was changed to T5201 (recognizes all five isoforms of beta-tubulin and binds to the carboxy-terminal part, Sigma). Sc-389837 was diluted to 45 nM in order to get the final concentration of 1 nM, T5201 to 135 nM to get final concentration of 3 nM, and ab6046 was diluted to 450 nM to get 10 nM in the final mix. All dilution was made in AlphaLISA Immunoassay Buffer (Perkin Elmer AL000F). Anti-rabbit acceptor beads (Perkin Elmer AL104M) and anti-mouse donor beads (Perkin Elmer AS104D) were diluted in the same AlphaLISA buffer to 22.5 µg/ml and 90 µg/ml respectively to get 10 µg/ml and 40 µg/ml as final concentration when adding 4 µl of the mix to each well. All handling of the beads was done in a room with green light. Plates were sealed with sticky film, covered in foil, and spun at 177 rcf for 1–3 min. After overnight incubation in the dark, plates were spun briefly at 300 rcf and read in an Enspire 2300 (Perkin Elmer).

### Conjugation of AlphaLISA beads

Two antibody pairs were conjugated: ab6046 (abcam) and T5201 (Sigma) for tubulin (pair 8) and the polyclonal AF3418 for SOD-1. The SOD-1 antibody AF3418 (R&D Systems) was divided in two aliquots of which one was biotinylated and one was conjugated to the Acceptor beads (Perkin Elmer 6772001). For β-tubulin ab6046 (abcam) was biotinylated and T5201 (Sigma) was used for conjugation to Acceptor beads. Biotinylation of respective antibody were performed using an antibody biotinylation kit (Pierce/Thermo scientific 90407).

Before performing biotinylation, the buffer of ab6046 needed to be exchanged to PBS which was done using Zeba spin column (Thermo Scientific 89882) according to the manufacture’s instructions. Then 100 µl PBS was added to one tube of NHS-PEG4-Biotin and mixed by pipetting. A 40-fold excess of biotin was added to the antibodies, mixed, and incubated for 30 min at RT. After incubation, the buffer was exchanged using the Zeba spin column (Thermo Scientific 89882) according to manufacturor’s protocol. Biotinylated antibodies were aliquoted and stored at −20 °C before use.

The antibodies T5201 and AF3418 were conjugated to Acceptor beads (Perkin Elmer 6772001). 0.033 mg beads were used for 0.333 mg antibody. First, the Acceptor beads were washed by adding 17 µl PBS (Life technologies 10010), centrifuged at 16 000 rpm for 15 min, supernatant discarded, and the pellet dissolved in at mix with 0.033 mg antibody and 130 mM sodium phosphate puffer (pH 8.0) in a final volume of 67 µl. In later conjugations, this volume was reduced to half in order to increase the yield. To this mix 0.43 µl of 10% Tween-20 (P1379) and 3.33 µl of 400 mM NaHB3CN (Sigma Aldrich 296945) was added, mixed gently by pipetting and incubated for 18–24 h at 37 °C with mild agitation (~60 rpm).

The next day 3.33 µl of 65 mg/ml Carboxymethoxylamine (Sigma C13408) in 800 mM NaOH was added and incubated for 1 h at 37 °C with mild agitation to stop the reaction. After incubation the tubes were centrifuged for 15 min at 16 000 rpm at 4 °C and the supernatant discarded. The pellet was resuspended in 67 µl 100 mM Tris-HCl (pH 8.0), centrifuged again and was resuspended in 200 µl PBS with 0.05% Proclin-300 (Sigma Aldrich 48912). The antibodies were vortexed briefly, spun down, and sonicated with 10 pulses before stored at + 4 °C until use.

### AlphaLISA assay with conjugated beads

Samples were diluted in HBSS for optimal linearity for each antibody pair. For human samples, 300 cells/well were used for SOD-1 and 3000 cells/well for β-tubulin. The PDX-samples were diluted 10 x for tubulin and 500 times for SOD1. 3 µl of each sample was loaded in AlphaPlate (Perkin Elmer 6008350), in duplicate when possible. 2 µl biotinylated antibody was added to each well and the plate were then briefly spun down before addition of Acceptor beads. For both β-tubulin and SOD-1 the biotinylated antibody (biotin-AF3418 and biotin-ab6046 respectively) were used at a final concentration of 3 nM.

Acceptor breads (2 µl) were added and the plate was spun briefly and incubated on a shaker for 1 h at 60 rpm. For SOD-1, Acceptor beads were diluted to 45 µg/ml for a final concentration of 10 µg/ml and for β-tubulin T5201-conjugated Acceptor beads were used at a concentration of 180 µg to get at final concentration of 40 µg/ml. Then 2 µl Donor beads (Perkin Elmer 6760002S) was added at a concentration of 180 µg/ml for get at final concentration of 40 µg/ml. Handling of beads was only done in a room with green light. Plates were sealed with sticky film, covered in foil, briefly spun down, and incubated in the dark over night before being read in an EnSpire 2300 (Perkin Elmer).

### ITDR-CETSA in multidrug-resistant K562 cells

For the intact cell ITDR-CETSA experiments, studied compounds (docetaxel, paclitaxel, or epothilone B) were prepared in RPMI supplemented with 5% FBS at double the final compound concentration and with or without the addition of tariquidar (also at double the final concentration). Multidrug-resistant and parental K562 cells were harvested, washed in + 5% FBS, and resuspended at 6,66 million cells/ml in RPMI + 5% FBS. Cells were then mixed 1:1 in PCR tubes with the previously prepared compound dilutions so that the final cell density was 3,33 million cells/ml in 50 µl total volume. The final tariquidar concentration in the allocated samples was 0,5 µM. Vehicle-treated samples were also included at a final concentration of 0,4% DMSO. The treated cells were incubated at 37 °C for 1 h 45 min followed by heating for 3 minutes at 63 °C in a Veriti thermal cycler (Applied Biosystems). Unheated samples were also included. Immediately after the heating, all samples were freeze-thawed three times using liquid nitrogen. The samples were vortexed after every freeze-thaw cycle. The remaining soluble β-tubulin was analyzed in the total lysate using AlphaLISA pair 8.

### Resazurin cytotoxicity assay

For assessment of drug toxicity, multidrug-resistant and parental K562 cells in duplicates of 10 000 cells/well, were seeded in black 96-well clear bottom polystyrene microplates (Sigma) containing various drug concentrations diluted in RPMI supplemented with 5% heat inactivated FBS. Following, 72 h incubation at 37 °C, 10 μg/ml resazurin sodium salt (Sigma) was added to each well and further incubated for 2 h at 37 °C. Resorufin fluorescence was measured at a wavelength of 590 nm using an Enspire plate reader (Perkin Elmer).

### Image-CETSA

Multidrug-resistant and parental K562 cells were propagated in RPMI-1640 medium supplemented with 10% heat inactivated FBS and continuously maintained at a cell density of 2 × 10^5^ cells/ml. Drug resistance was preserved with 150 nM vincristine or vehicle DMSO, in the resistant and parental K562 cells respectively, by selection for 72–96 h. Prior to each experiment, cells were washed once in HBSS before transfer to PCR tubes with RPMI-1640 medium containing 5% heat inactivated FBS and various drug concentrations at a density of 4 million cells/ml. After 2 h incubation at 37 °C, CETSA heating was performed at 56 °C in a Veriti Thermal Cycler (Applied Biosystems) for 3 min. After heating, cells were collected by centrifugation at 200 rcf for 3 min at 4 °C and washed twice in HBSS before transfer to black 96-well clear bottom polystyrene microplates (Sigma) pre-coated with 1% aqueous Alcian Blue solution (Electron Microscopy Sciences) for 15 min for cell adherence. Supernatant was aspirated after centrifugation at 200 rcf for 5 min at 4 °C, after which cells were fixed in 4% w/v formaldehyde (Thermo Scientific) for 15 min at room temperature. Next, the cells were washed twice in DPBS/Modified (GE Healthcare) and permeabilized by incubation with 5% v/v Triton X-100 (Sigma) for 15 min at room temperature with gentle agitation. After an additional three washes, blocking was performed in 2% w/v bovine serum albumin (Sigma) for 1 h at room temperature with gentle agitation. β-tubulin conjugated Alexa Fluor 555 (#3623, Cell Signaling) was diluted 1:200 in blocking buffer and added for overnight incubation protected from light at 4 °C with gentle agitation. Next, the cells were washed twice with 0.1% v/v Tween 20 (Sigma) and counterstained with 1 mg/ml v/v Hoechst (Chemometec) diluted in DPBS/Modified for 5 min at room temperature with gentle agitation. After an additional two washes, the Tween 20 wash buffer was replaced with DPBS/Modified for subsequent image analysis.

Images and analysis were acquired with the Cytell^TM^ Cell Imaging system (GE Healthcare) using a 2-colour BioApp specific for cytoplasmic image analysis. A blue (390/430) and a yellow (544/588) channel were applied for nuclear and cytoplasmic imaging respectively. Fifty-one fields covering the entire well were captured with a 10x Plan Apo 0.45 NA objective. Raw data based on average cellular cytoplasmic intensity for each individual well was further analyzed and plotted using GraphPad Prism version 6.

### CETSA on *in vivo* treated xenografts

Female NOD-SCID mice were obtained from the breeding unit at the Department of Microbiology, Tumor and Cell Biology, Karolinska Institute. All mouse studies were approved by the Northern Stockholm Experimental Animal Ethical Committee (Dnr N 192/13 and Dnr N 2/17) and were performed in accordance with the relevant guidelines and regulations. Tumour volumes were measured with a caliper and calculated according to the standard formula (length × width^2^ × 0.52).

For the *in vivo* experiments, NOD-SCID mice were injected with approximately 5 × 10^6^ MCF-7 cells into a mammary gland. When the tumor sizes reached 0.2–0.3 cm^3^, the mice were injected with either vehicle (EtOH 1:1 in polysorbate 80 and then diluted in 5% dextrose) or docetaxel 30 min before they were euthanized by inhalation of a lethal dose of CO_2_ followed by cervical dislocation. Tumours were removed and cut into pieces using a scalpel and put in PCR-strips (Applied Biosystems N8010580). 30 µl HBSS (Life technologies 14025100 + CaCl_2_, + MgCl_2_) was added and the samples heated to different temperatures in a Veriti Thermal cycler (Applied Biosystems). After heating samples were frozen in liquid nitrogen and then thawed at 20 °C. High salt lysis buffer (50 mM tris-HCl, 300 mM NaCl, 0.5% NP40, 1 mM EDTA, pH 7.2) and Halt Protease Inhibitor (Thermo Scientific 1861279) was added and the freeze-thawing repeated two additional times. The tumour pieces were further homogenized by crushing them with bent plastic pipette tips.

Soluble proteins were isolated by centrifugation at 20000 x g for 20 minutes at 4 °C and the tubulin detected with western blot or AlphaLISA pair 2. SOD-1 was used as loading control in order to minimize the potential effect of differences in size of tumour pieces, this did however only give minimal change to the graph appearance, why we chose not to use this in the analysis of xenografts. Total five animals were use for the first pilot experiment and 16 mice were used for the second.

### CETSA on *ex vivo* treated xenografts

NOD-SCID mice were injected with approximately 5 million MDA-MD-231 cells into a mammary gland. At the day of the experiment, four mice were euthanized by inhalation of a lethal dose of CO_2_ followed by cervical dislocation and tumours removed directly. Each tumour was divided into 12 pieces, which were incubated in vehicle (DMSO, 0.5%) or 50 µM docetaxel in HBSS (Life technologies 14025100 + CaCl_2_, + MgCl_2_) supplemented with Halt Protease Inhibitor (Thermo Scientific 1861279) for 1 h at 37 °C. After incubation samples were heated to different temperatures in a Veriti Thermal cycler (Applied Biosystems). After heating samples were frozen in liquid nitrogen and then thawed at 20 °C. High salt lysis buffer (50 mM tris-HCl, 300 mM NaCl, 0.5% NP40, 1 mM EDTA, pH 7.2) was added and the freeze-thawing repeated two additional times. The tumour pieces were further homogenized by crushing them with bent plastic pipette tips. Soluble proteins were isolated by centrifugation at 20000 rcf for 20 minutes at 4 °C and tubulin was detected with western blot.

### CETSA on *ex vivo* treated PDX-tumours

These animal experiments were performed at the Erasmus University Medical Centre in Rotterdam and all experiments were approved by the Animal Experiment Committee under the Dutch Experiment on Animals Act and adhered to the European Convention for Protection of Vertebrate Animals used for Experimental Purposes (Directive 2010/63/EU).

Sixteen mice were inoculated with one of four prostate cancer cell lines bilaterally at 5 million cells on each side. The following prostate cancer cell lines were used: PC346C and PC339, and the docetaxel resistant lines PC346C-Doc and PC339-Doc. Cells were maintained as described previously^37^.

Tumour volume was monitored twice weekly by digital calipers, and tumours were excised 28–37 days after inoculation (tumour volumes ranged between 125 and 1500 mm^3^).

At the day of the experiment, mice were sacrificed (cervical dislocation), the tumours removed and fixed on a layer of low melting agarose using cyanoacrylate glue. The tumours were sliced using the Leica VT1200S Vibratome at a thickness of 300 µm and the slices were transferred to PCR strips containing HBSS (Life technologies 14025100 + CaCl_2_, + MgCl_2_) and treated with a range of drug concentrations as indicated for 1 h at 37 °C and 5% CO_2_. After incubation, samples were heated in a PCR-machine (Biometra T1 thermocycler) at 63 °C for 3 min and then snap frozen in liquid nitrogen. All samples were stored at −80 °C until detection.

For detection, all samples were frozen and thawed three times in liquid nitrogen and a 20 °C program on a PCR-machine. High salt lysis buffer (50 mM tris-HCl, 300 mM NaCl, 0.5% NP40, 1 mM EDTA, pH 7.2) was added and samples were vortexed vigorously before slices and debris were removed by centrifugation for 20 min at 1000 rpm. Supernatant was isolated and analyzed for β-tubulin and SOD-1 content using AlphaLISA. For β-tubulin detection antibody pair 2 was used and the samples were diluted 10 times prior to AlphaLISA detection, while for SOD-1 samples were diluted 500 times.

### Patient fine needle aspirates (FNAs)

All experiments performed on human material were approved by the Regional Ethical Board in Stockholm. Samples were either anonymized (Dnr 2016 957-31) or taken from patients who signed informed consent (Dnr 2015/1694-31/1 with approved amendments 2016/2599-32, 2017/1353-32 and 2017/1267-32). All methods were performed in accordance with the relevant guidelines and regulations. FNAs were taken by pathologists or cytologists from tumours after surgery or directly from patients and placed in tubes with L15 media (Gibco L-15 21083-027). Cells were counted, spun down, and resuspended in HBSS (Life technologies 14025100 + CaCl_2_, + MgCl_2_) and treated with docetaxel for 15 min. When 100 000 cells or more were obtained, cells were resuspended to a final concentration of 3 million cells/ml and detected with AlphaLISA pair 8. When 20 000 to 100 00 cells were obtained cells were suspended to 1 million/ml and detected with conjugated AlphaLISA pair 8.

### Data analysis

The presented data were generated in independent experiments n ≥ 3 except Figs. 2C–E, S2D–F and Fig. 7, where data correspond to technical duplicates. All graphs were generated using GraphPad Prism (versions 6–8). All data are presented as mean with error bars representing the standard error of the mean (S.E.M). Error bars that are smaller than the displayed data points are not shown by the software e.g. in Fig. 2C.

ITDR-CETSA and cell viability data are presented as a percentage of the signal corresponding to the compound concentration where maximum stabilization was achieved in each series, with the exception of Figs. 6E and S5E where data are presented as normalized to SOD-1, Fig. 6B–D where data are presented as a percentage of the signal obtained for the drug-sensitive cells at maximum stabilization, and Fig. 7B,C where data are presented as a percentage of the signal detected for the untreated samples in each series. Sigmoidal curves were fit in GraphPad Prism using non-linear regression of the type [Inhibitor] vs. Response (three parameters) with the function Y = Bottom + (Top-Bottom)/(1 + (X/IC50)). ITDR-CETSA curves in Fig. 7 were fit using non-linear regression of the type [Inhibitor] vs. Response (four parameters) with the function Y = Bottom + (Top-Bottom)/(1 + (IC50/X)^HillSlope). The EC_50_ values with associated standard errors and 95% confidence intervals calculated for the curves are presented in corresponding tables. The statistical significance of the difference observed between the dose-response curves in each graph was calculated using a T-test for the EC_50_ values obtained in each individual experiment.

For CETSA melt curves (Figs. 1B–G, 2C,D, 4A and 5A–C), data are presented as a percentage of the signal detected at the lowest temperature in each melt curve and a line connecting the data points was automatically generated by the abovementioned software. Melting temperatures (Tm) and the size of the CETSA shifts (ΔTm) were also determined in GraphPad Prism. One-way ANOVA was used for analyzing the CETSA melt curve data in Figs. 1B–G and 5A–C. The significance levels for these statistical analyses are presented in corresponding tables.

## Supplementary information

Supplementary Information

## Data Availability

The datasets generated and analysed during the current study are available from the corresponding author on reasonable request.
