# Supplementary material for: CETSA-based target engagement of taxanes as biomarkers for efficacy and resistance
Source: Sci Rep. 2019 Dec 18;9:19384. doi: 10.1038/s41598-019-55526-8 (PMC6920357; doi:10.1038/s41598-019-55526-8)
Supplement: Supplementary file 1 — Supplementary Information [file 41598_2019_55526_MOESM1_ESM.pdf]

CETSA-based target engagement of taxanes as biomarkers for efficacy and resistance

Anette Langebäck[1,¶], Smaranda Bacanu[1,¶], Henriette Laursen[1,¶], Lisanne Mout[4,¶], Takahiro Seki[5], Sigrun Erkens-Schulze[4], Anderson Daniel Ramos[1], Anna Berggren[1], Yihai Cao[5], Johan Hartman[1], Wytske van Weerden[4], Jonas Bergh[1], Pär Nordlund[1,2,3,*] and Sara Lööf[1]

1. Department of Oncology and Pathology, Karolinska Institutet, Stockholm, 17177, Sweden
2. School of Biological Sciences, Nanyang Technological University, 637551, Singapore
3. Institute of Molecular and Cell Biology, A*STAR, 138673, Singapore
4. Department of Urology, Erasmus Medical Centre, Rotterdam, The Netherlands
5. Department of Microbiology, Tumour and Cell Biology, Karolinska Institutet, Stockholm, 17177, Sweden

¶ These authors contributed equally to this work

* Corresponding author: E-mail: par.nordlund@ki.se (Pär Nordlund)

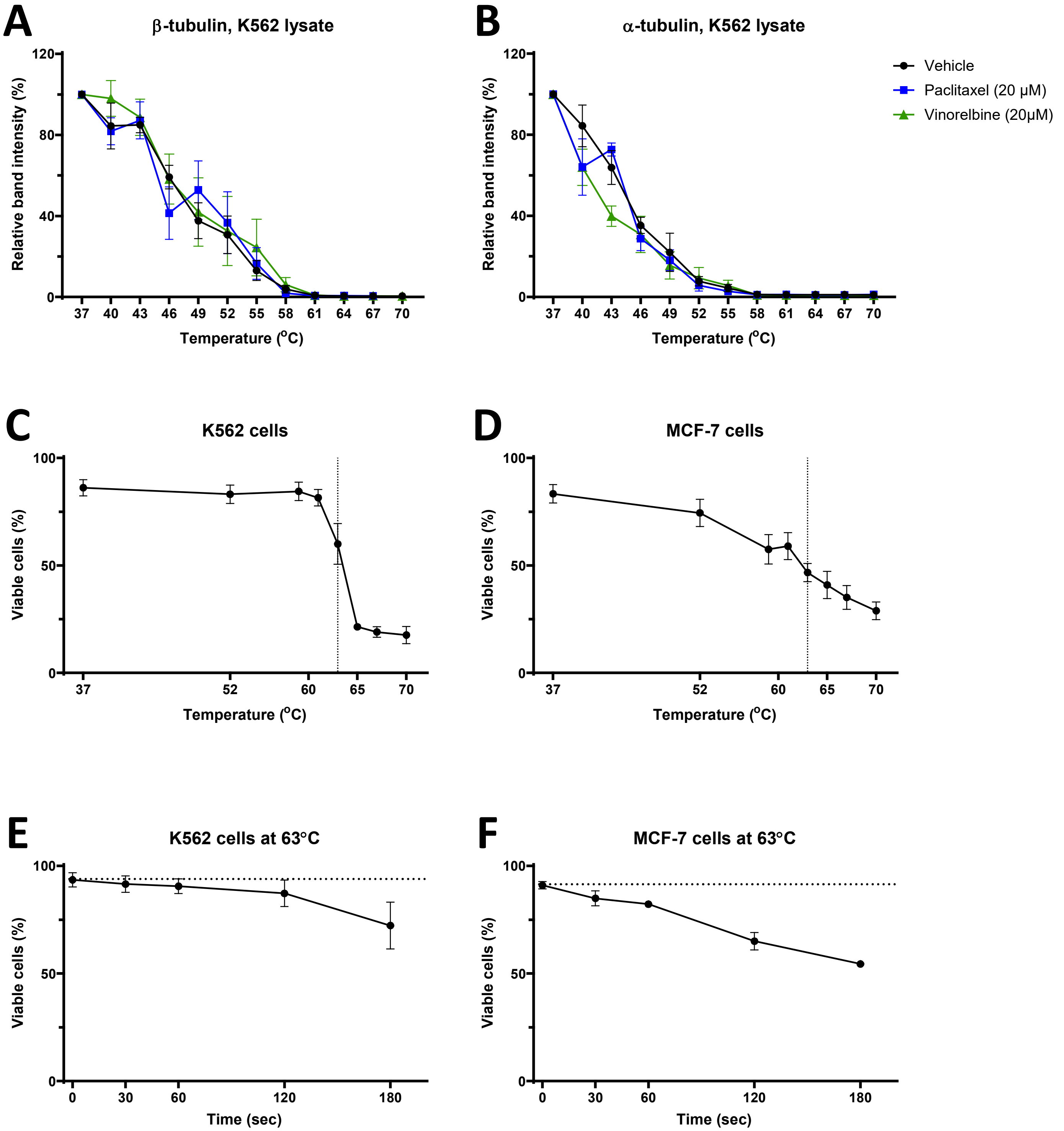

Figure S1. CETSA melt curves for α-tubulin and β-tubulin in K562 lysate treated with paclitaxel or vinorelbine (20 μM) detected with western blot (A and B). Membrane integrity detected with trypan blue exclusion in K562 (C) and MCF-7 cells (D) upon 3 min heating at different temperatures. Membrane integrity detected after heating to 63 °C for various time points in K562 (E) and MCF-7 cells (F).

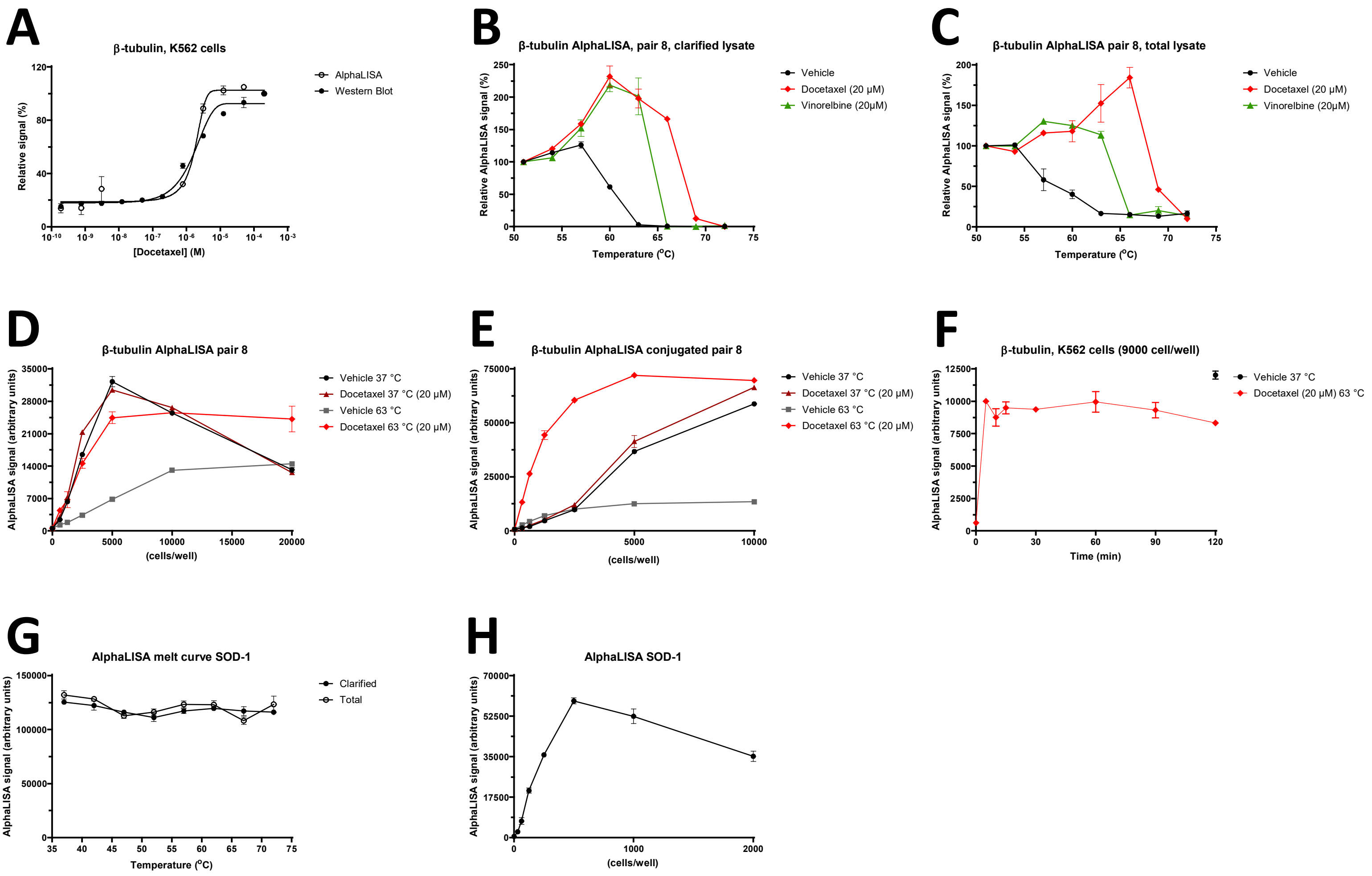

Figure S2. Comparison of ITDR-CETSA at 64 °C detected with β-tubulin AlphaLISA pair 2 and western blot (A). CETSA-melt curve for β-tubulin after treatment with docetaxel or vinorelbine detected with pair 8 in (B) clarified or total lysate (C). Different amounts of cells treated with docetaxel or vehicle were detected with standard AlphaLISA pair 8 (D) or conjugated pair 8 (E), showing the range and linearity for these methods. β-tubulin stabilization detected with AlphaLISA pair 2 after different time points of drug treatment (F). SOD-1 was used as loading control for some experiments due to its stability during heating (G). Linearity for the SOD-1 AlphaLISA assay (H).

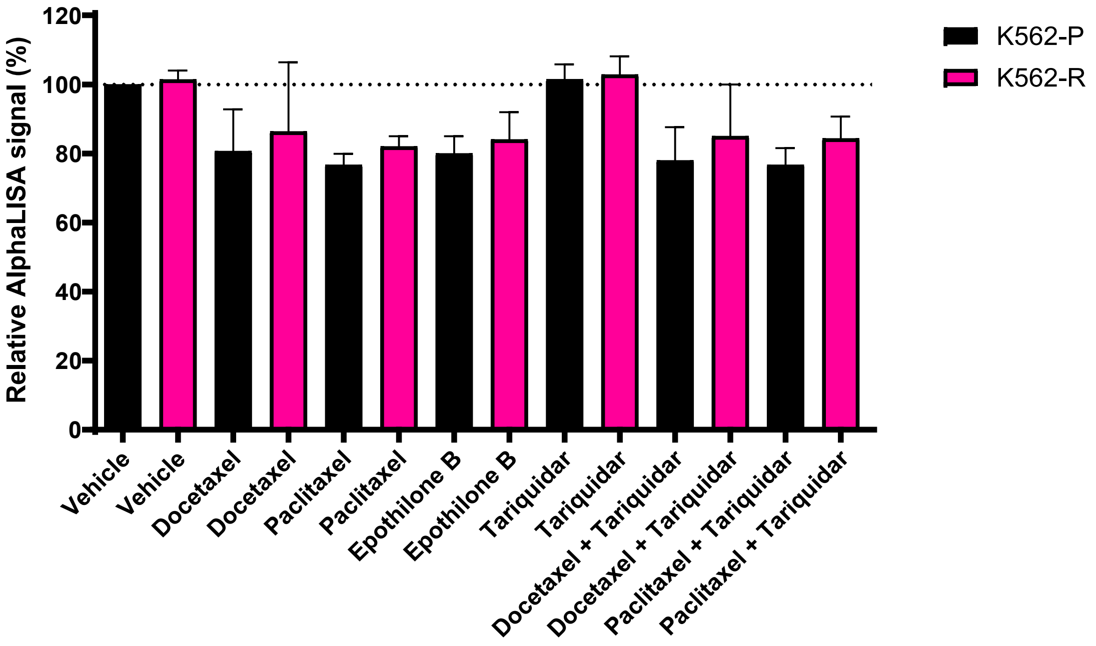

Figure S3. Levels of β-tubulin detected with AlphaLISA in parental (K562-P) and resistant (K562-R) cells with and without exposure to the tubulin-binding drugs (100 μM) and tariquidar (0.5 μM).

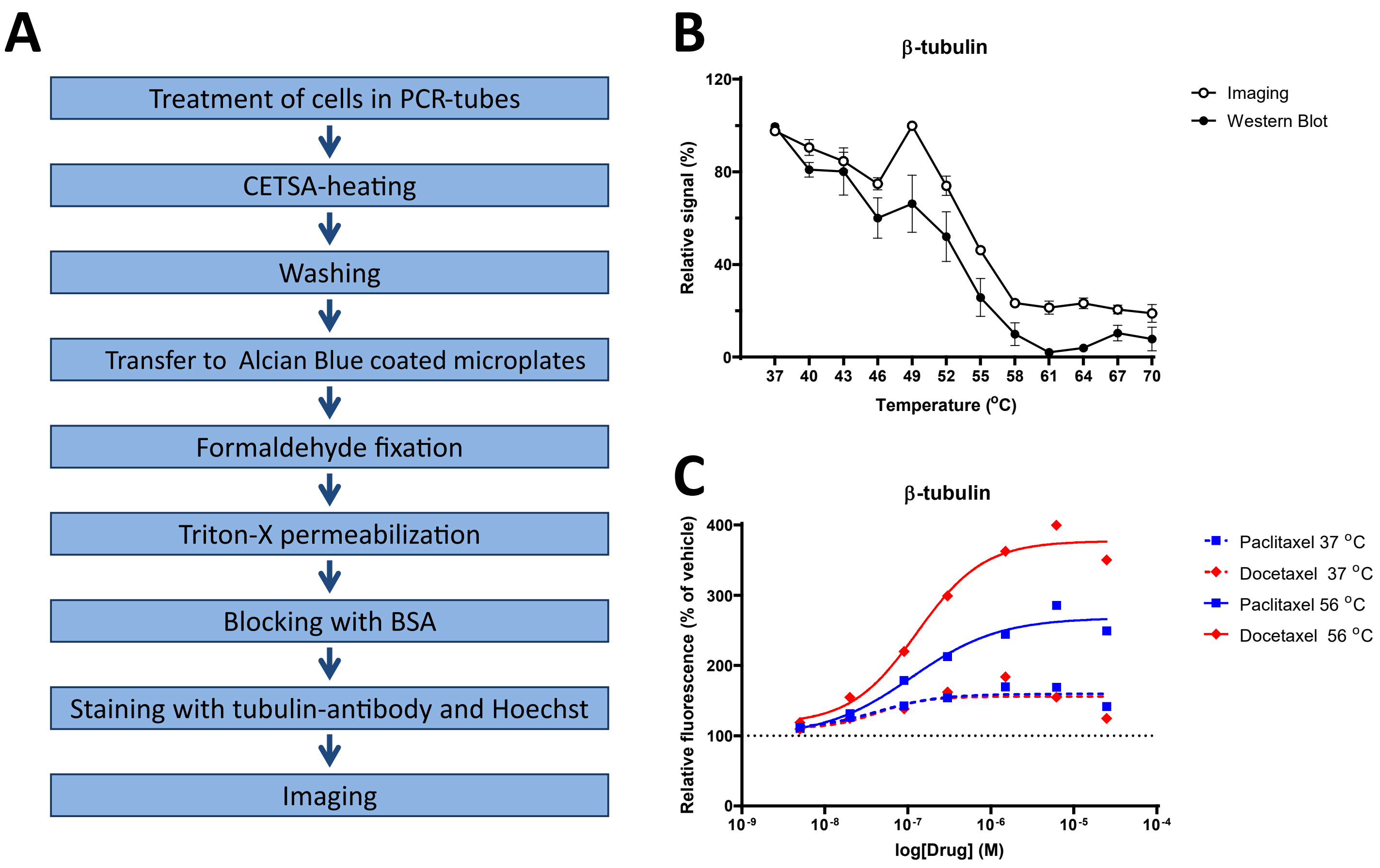

Figure S4. CETSA data achieved by Imaging and WB report similar melting behavior for β-tubulin and control ITDRs run at 37°C and detected with Image-CETSA produce no dose-response curves.

Overview of the protocol used for imaging CETSA (A). CETSA melt curves in K562 cells show similar melting pattern for β-tubulin when detected with cell imaging or western blot (B). CETSA response to increasing taxane concentrations in K562 heated to 37 °C or 56 °C (C). At 37 °C the taxanes produce a minimal increase in fluorescence while at 56 °C a dose-dependent response was detected for both paclitaxel and docetaxel.

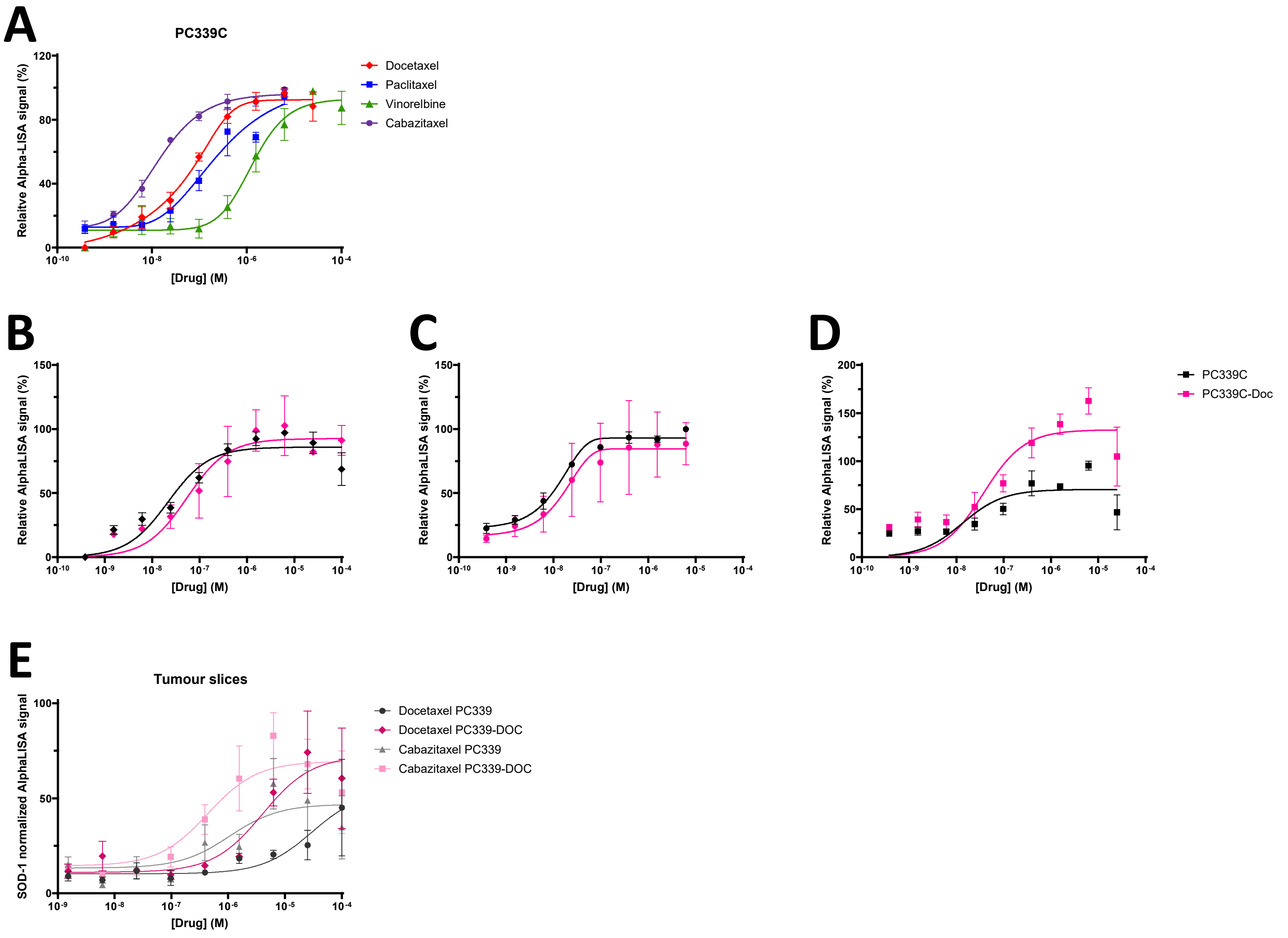

Figure S5. CETSA TE in mouse PDX models of prostate cancer.
PDX-derived cell line PC339 was treated with different concentrations of tubulin-binding drugs and β-tubulin TE was analysed with AlphaLISA (A) PC339C cells and the corresponding resistant cell line PC339C-DOC were treated with different concentrations of taxanes (B-D). Tumours from the PDX model PC339 and the resistant counterpart PC339-DOC were treated *ex vivo* with increasing doses of docetaxel and cabazitaxel (E). Data from tumours slices were normalized to SOD-1 levels. All data represent the mean ±S.E.M from either different tumors in each condition (E) or from independent experiments (n=3 in A-D).

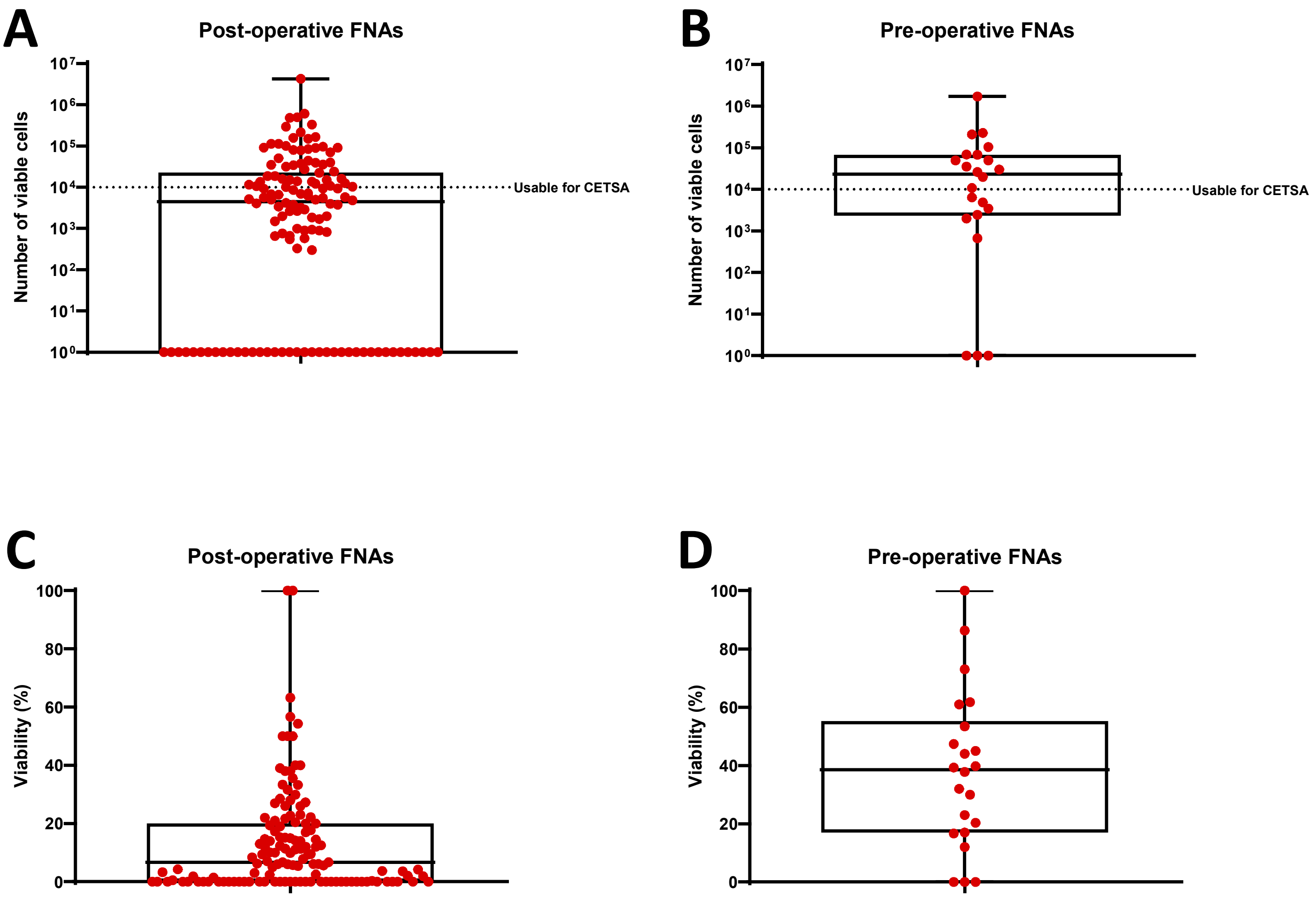

Figure S6. Variation in FNA sample viability.
Number of viable cells in FNAs from surgically removed tumors (A) or FNAs taken directly from patients (B). Ratio of viable cells in FNA-samples taken after surgery (C) and in FNAs taken directly from patients (D).

Table S1A. Characteristics of the PDX models used for generating the data in Fig 6 and FigS5

	**PC339**		**PC339-Doc**		**PC346C**		**PC346C-Doc**	
	Doc	Cab	Doc	Cab	Doc	Cab	Doc	Cab
**Tumour Volume***	-	+++	-	+++	+	+	-	-
**Drug Concentration (ng/mg)****	0,2	4	0,2	8	2	3	n/a	n/a
**Proliferation**	No	Yes			Yes	Yes		

*Effect of drug on tumour volume. **Drug concentration in tumour after 7 days.

Table S1B. Overview of EC_{50} values with associated standard errors and 95% confidence intervals (CI) calculated for the curves in Fig 6A and S5A.

**CETSA**	**PC346C**		**PC339C**	
	EC50 and St. Error (µM)	95% CI	EC50 and St. Error (µM)	95% CI
Docetaxel	0,08±0,02	0,04-0,15	0,07±0,01	0,04-0,10
Paclitaxel	0,13±0,03	0,08-0,2	0,14±0,05	0,07-0,31
Cabazitaxel	0,01±0,007	0,005-0,04	0,01±0,002	0,009-0,02
Vinorelbine	2,02±0,31	1,47-2,78	1,40±0,44	0,75-2,61

Table S1C. Overview of EC_{50} values with associated standard errors and 95% confidence intervals (CI) calculated for the curves in Fig 6E and S5E.

	**CETSA**	
**Docetaxel**	EC50 and St. Error (µM)	95% CI
PC346C	0,85±0,60	0,26-2,74
PC346C-DOC	n/a	n/a
PC339	30,00±39,40	0-111
PC339-DOC	4,01±2,81	0-9,95
**Cabazitaxel**	EC50 and St. Error (µM)	95% CI
PC346C	1,48±1,00	0,30-6,00
PC346C-DOC	1,00±1,13	0,12-5,21
PC339	1,06±1,28	0-3,71
PC339-DOC	0,44±0,38	0-1,24

Table S1D. Overview of EC_{50} values with associated standard errors and 95% confidence intervals (CI) calculated for the curves in Fig 6B-D.

	**PC346**		**PC346-DOC**	
**CETSA**	EC50 and St. Error (µM)	95% CI	EC50 and St. Error (µM)	95% CI
Docetaxel	0,06±0,02	0,03-0,13	0,70±0,28	0,30-1,5
Cabazitaxel	0,01±0,005	0,004-0,03	0,06±0,04	0,02-0,21
Paclitaxel	0,12±0,03	0,047-0,20	n/a	n/a

Table S2. Overview of EC_{50} values with associated standard errors and 95% confidence intervals (CI) calculated for the ITDR-CETSA curves in Fig 7 B and C.

**FNAs**	**β-tubulin**	
**Post-operative**	EC50 and St. Error (μM)	95% CI
Patient 1	4,00±0,11	2,80-5,80
Patient 2	0,24±0,07	0,10-0,44
Patient 3	0,60±0,14	0,24-1,07
Patient 4	n/a	n/a
Patient 5	n/a	n/a
Patient 6	0,20±0,01	0,06-0,50
**Pre-operative**		
Patient A	n/a	n/a
Patient B	n/a	n/a
Patient C	n/a	n/a
Patient D	0,64±0,33	0,30-1,53
Patient E	n/a	n/a
